# p62/Sequestosome-1 Is Indispensable for Maturation and Stabilization of Mallory-Denk Bodies

**DOI:** 10.1371/journal.pone.0161083

**Published:** 2016-08-15

**Authors:** Pooja Lahiri, Volker Schmidt, Claudia Smole, Iris Kufferath, Helmut Denk, Pavel Strnad, Thomas Rülicke, Leopold F. Fröhlich, Kurt Zatloukal

**Affiliations:** 1 Institute of Pathology, Medical University of Graz, Graz, Austria; 2 Institute of Laboratory Animal Science, University of Veterinary Medicine Vienna, Vienna, Austria; 3 IZKF and Department of Internal Medicine III, Aachen, Germany; Indian Institute of Science Education and Research, INDIA

## Abstract

Mallory-Denk bodies (MDBs) are hepatocytic protein aggregates found in steatohepatitis and several other chronic liver diseases as well as hepatocellular carcinoma. MDBs are mainly composed of phosphorylated keratins and stress protein p62/Sequestosome-1 (p62), which is a common component of cytoplasmic aggregates in a variety of protein aggregation diseases. In contrast to the well-established role of keratins, the role of p62 in MDB pathogenesis is still elusive. We have generated total and hepatocyte-specific p62 knockout mice, fed them with 3,5-diethoxycarbonyl-1,4-dihydrocollidine (DDC) to induce MDBs and allowed the mice to recover from DDC intoxication on a standard diet to investigate the role of p62 in MDB formation and elimination. In the absence of p62, smaller, granular and less distinct MDBs appeared, which failed to mature to larger and compact inclusions. Moreover, p62 deficiency impaired the binding of other proteins such as NBR1 and Hsp25 to MDBs and altered the cellular defense mechanism by downregulation of Nrf2 target genes. Upon recovery from DDC intoxication on a standard diet, there was an enhanced reduction of p62-deficient MDBs, which was accompanied by a pronounced decrease in ubiquitinated proteins. Our data provide strong evidence that keratin aggregation is the initial step in MDB formation in steatohepatitis-related mouse models. Interaction of p62 with keratin aggregates then leads to maturation i.e., enlargement and stabilization of the MDBs as well as recruitment of other MDB-associated proteins.

## Introduction

The fate of misfolded proteins gained significant relevance in a variety of diseases characterized by the accumulation of abnormal proteins, collectively known as ‟protein aggregation diseases” [1, 2]. The folding of proteins into their functionally active three-dimensional structures is among the most fundamental roles of a living cell [2]. Misfolding on genetic or toxic basis may lead to aggregation-prone proteins, being deposited in the cytoplasm, nucleus or extracellular compartments [1, 3]. These inclusion bodies can serve as morphological hallmarks of a variety of diseases. They include among others, neuronal Lewy bodies in Parkinson’s disease, neurofibrillary tangles in Alzheimer’s disease, TDP-43 aggregates in amyotrophic lateral sclerosis, and Mallory-Denk bodies (MDBs) in steatohepatitis as well as other chronic liver disorders [3–5]. As a reflection of a common pathogenesis, these inclusions display, in addition to their disease-specific protein ‟backbone”, a common molecular composition, since they collectively contain the multifunctional stress and adaptor protein p62/Sequestosome-1 (p62) and ubiquitin (Ub) as constant constituents.

In this study, we focused on the function of p62 in the formation of MDBs *in vivo*. MDBs are hepatocytic protein inclusions found in several chronic liver diseases, especially alcoholic (ASH) or non-alcoholic (NASH) steatohepatitis, idiopathic copper toxicosis, chronic cholestasis, and hepatocellular carcinoma (HCC) [5]. MDBs can be induced in mice by chronic treatment with chemicals such as griseofulvin or 3,5-diethoxycarbonyl-1,4-dihydrocollidine (DDC) [5, 6]. Of note, both human and murine MDBs are reversible; they disappear in ASH after alcohol cessation or in mice upon recovery on a standard diet after prolonged DDC intoxication [7].

MDBs mainly consist of cytoskeletal intermediate filament proteins keratin 8 (K8) and keratin 18 (K18), p62 and ubiquitin [5, 8, 9]. Furthermore, they contain a high molecular weight component that is recognized by the M_M_120-1 antibody. This component has so far been exclusively detected in human and murine MDBs and, therefore, serves as a specific marker of MDBs [10]. In addition to these major MDB constituents, several proteins have been found as facultative MDB components, such as heat shock proteins 25 and 70 (Hsp25 and Hsp70) and the structural homolog of p62 called Neighbor of BRCA1 (NBR1) [5, 11]. In MDBs, keratins are hyperphosphorylated, transamidated, and partially degraded [12–15]. Moreover, keratins (K8 in particular) exhibit a conformational change from predominantly α-helical to cross β-sheet (amyloid-like) structure [14, 16]. Earlier *in vitro* studies on MDB formation showed that p62 binds to ubiquitinated, misfolded keratins [9, 17, 18]. It has been previously reported that p62 binds polyubiquitinated proteins via its UBA (ubiquitin associated) domain, shapes them into sequestosomes and shuttles them to degradation pathways, such as the ubiquitin proteasomal system (UPS) for ubiquitinated and soluble proteins or the autophagic machinery, which is responsible for bulk degradation of larger structures, such as protein aggregates or organelles [19–21]. Also, p62 itself can form aggregates such as intracellular hyaline bodies (IHBs) in livers of patients with idiopathic copper toxicosis and HCC [22]. In these liver diseases, transition stages between keratin-negative IHBs and keratin-positive MDBs have been observed [22, 23]. This led to the conclusion that aggregates of p62 might serve as a matrix for the incorporation of ‟abnormal” keratins eventually leading to MDB formation [18]. Whether this mechanism is also relevant to MDB pathogenesis in ASH, NASH or related animal models is not clear [24, 25]. It has been previously argued that soluble oligomeric proteins are detrimental to cells [1], and p62 may play a protective role by packing them into insoluble and less toxic aggresomes or sequestosome-like aggregates [26]. In addition to its role in MDB formation, p62 may also be involved in the elimination of MDBs. It has been suggested that MDBs are degraded by the UPS and autophagy, and p62 may play a role in both processes [27]. Although aggregation properties of p62 have been intensively investigated in cell culture experiments, no data exist on p62’s *in vivo* role in disease-related protein aggregates. Since p62 is expressed in most, if not all, cell types and has been shown to play a role in a variety of cellular processes, such as fat metabolism, autophagy, and cell proliferation [28, 29], we had to distinguish between cell autonomous roles of p62 in MDB formation in hepatocytes from possible indirect effects mediated by other cells (e.g., by bile duct epithelia, macrophages, inflammatory cells) or systemic metabolic effects. Therefore, we have generated p62 total (*p62*^*-/-*^)-and hepatocyte-specific (*p62hep*^*-/-*^)-knockout mice and intoxicated them with DDC to induce MDBs to delve into the *in vivo* role of p62 in MDB formation.

## Materials and Methods

### Generation of Total (*p62*^*-/-*^) and Hepatocyte-Specific (*p62hep*^*-/-*^) p62-Knockout Mice

We generated *p62-*knockout mice using the Cre/loxP strategy. 5' fragment of *p62/Sqstm1* gene (NC_000077) containing exons 1–5 was PCR-amplified and inserted via SacI into the pBluescript vector (kindly provided by C. Birchmeier; Max-Delbrueck-Centre for Molecular Medicine, Berlin, Germany) upstream of the loxP-neomycin-loxP cassette. An additional loxP site was introduced before exon 5 using the QuickChange Site-Directed Mutagenesis protocol (Stratagene, La Jolla, CA). To complete the construct, the PCR-amplified 3' fragment of *p62/Sqstm1* gene was ligated via SalI into the vector and the BamHI-SalI-cleaved product was introduced into a wildtype ES (embryonic stem) cell line with a 129S2/SvPas background by electroporation. Neomycin resistant colonies were screened for homologous recombination by long-range PCR and the positive clones with a single integration event were microinjected into C57BL/6 blastocysts. Chimeric males were mated with C57BL/6-Tg(Meu-Cre40) animals [30, 31] to obtain mice which either lacked the neo-selection cassette but contained floxed exons 1 to 4 of the *Sqstm1* gene (*Sqstm1*^*tm1Biat*^, designated in the text as *p62*^*floxed/floxed*^ or *p62*^*f/f*^) or lacked the complete floxed region (*Sqstm1*^*tm1*.*1Biat*^, designated in the text as *p62*^*-/-*^) (S1 Fig). To generate a hepatocyte-specific *p62*-deficient mouse line (*p62hep*^*-/-*^), *p62*^*f/f*^ mice were mated with mice that expressed Cre-recombinase under the control of the albumin promoter (Alb-Cre) (S1 Fig) [30]. To obtain mice that were more susceptible to MDB formation, animals were crossed for several generations with wildtype 129S2/SvPas mice.

Genotyping was performed by PCR using genomic tail tip DNA and a combination of three sets of primers as shown in S1B and S1C Fig and S1 and S2 Tables. Littermate controls of the *p62*^*-/-*^ and *p62hep*^*-/-*^ mouse strains were included in all experiments. To test for the presence of the Alb-Cre transgene, an additional Cre-specific PCR was performed (S1C Fig; S1 Table, PCR C). The PCR conditions maintained were (i) initial denaturation at 95°C for 5 minutes, (ii) denaturation at 95°C for 30 sec (iii) annealing at 60°C for 30 seconds, (iv) elongation at 72°C for 30 seconds for 35 cycles and (v) Final elongation at 72°C for 10 minutes.

All animals used in this study were generated in Institute of Laboratory Animal Science, University of Veterinary Medicine Vienna, Vienna, Austria.

### Animal Experiments

MDBs were induced in two months old male *p62*^*-/-*^, *p62hep*^*-/-*^ and their corresponding controls *p62*^*f/f*^ and *p62hep*^*-/-*^ mice by feeding DDC (0.1% DDC wt/wt in standard diet; Sigma Aldrich, Steinheim, Germany) for 8 weeks [18]. To study MDB disappearance, DDC-fed mice were allowed to recover for 4 weeks on standard diet. At least five male mice per genotype and treatment condition were chosen for the experiments. Mice were sacrificed by cervical dislocation and blood was collected by cardiac puncture. To test for the serum parameters of liver damage, serum was separated by centrifuging the blood sample for 15 minutes at 2000 x g. Then, the serum was diluted in saline (1:3) to obtain values of seven different serum parameters from a single run of automatic chemistry analyzer (Hitachi, Roche, Switzerland). Livers were immediately removed and cut into pieces which were (i) snap frozen in methyl butane precooled with liquid nitrogen for immunofluorescence staining, (ii) fixed in 4% formaldehyde solution for histological/immunohistochemical analysis or (iii) placed in liquid nitrogen for biochemical/RNA expression analyses. Animal experiments were approved by the Austrian Federal Ministry of Science, Research and Economy in compliance with the Austrian Law for Welfare of Laboratory Animals with the license number: BMWF-66.010/0114-II/3b/2012 and BMWF-66.010/0114-ii/3b/2013. All animals received humane care according to the criteria outlined in the ‟Guide for the Care and Use of Laboratory Animals” prepared by the National Academy of Sciences and published by the National Institutes of Health (NIH publication 86–23 revised 1985).

### Extraction of RNA and Polymerase Chain Reactions

RNA extraction and cDNA preparation from livers were performed as described previously [18]. To check the efficiency of *p62* deletion, reverse transcriptase PCR (RT-PCR) was performed (S1 Table). To determine the expression of selected genes, quantitative real-time PCR (qPCR) was performed as described previously [18]. Samples were analyzed in triplicates using specific primers (S2 Table). 18S rRNA or β-actin was used as internal control. The amplification efficiencies for the genes of interest and the internal control were approximately equal, the quantification was expressed as a ratio of 2^∆Ct/gene^ to 2^∆Ct/internal control^, in which ∆Ct/gene and ∆Ct/internal control represent the difference between the threshold cycle of amplification for the gene of interest and internal control, respectively.

### Immunofluorescence and Immunoelectron Microscopy

Immunofluorescence staining was performed as described previously [18] with antibodies to target proteins (S3 Table). Proteins with cross β-sheet structure were detected with the luminescent conjugated oligothiophene h-HTAA [16]. Liver sections were analyzed with a LSM 510 META confocal laser scanning microscope (Carl Zeiss, Oberkoechen, Germany) with Plan-Neofluar 40/0.75 objective and a 32-element photomultiplier tube array detector (META detector accessory). Images were processed with Zeiss LSM Image Browser 4.2 software (Carl Zeiss, Oberkoechen, Germany). Immunoelectron microscopy was performed as described previously using acetone-fixed cryosections and M_M_120-1 antibody [17].

### Light Microscopy and Immunohistochemistry

Hematoxylin and eosin (H&E) and immunohistochemical staining’s for K8/K18 and p62 were performed on 4μm thick sections of formaldehyde-fixed paraffin-embedded (FFPE) liver tissue as previously described [22, 32].

### Protein Isolation and Western Blot

Whole liver tissue extracts were prepared using 3% sodium dodecyl sulphate (SDS)-containing buffer. Alternatively, soluble proteins were extracted with Triton-X-100 buffer and keratin-enriched fractions were obtained as described previously taking advantage of their relative insolubility in Triton-X-100 and high-salt buffers [33]. Nuclear fractions were extracted using subcellular protein fractionation kit (Thermo Scientific, Vienna, Austria). All western blots were performed on whole tissue extracts unless mentioned otherwise. Protein concentration was quantified by Bradford assay. Equal amounts of proteins were separated using Nu-PAGE Bis-Tris Gels (Life Technologies, Vienna, Austria) and transferred onto a PVDF membrane for immunoblotting with antibodies for target proteins (S3 Table). The antigen-antibody complexes were visualized using Pierce ECL Western Blotting Substrate (Thermo Scientific, Vienna, Austria).

### Data Analysis

The graphs were prepared and statistical analysis was performed using Prism 5 software (Graph Pad software, La Jolla, CA, USA). All reported p-values are two-tailed and values less than 0.05 were considered statistically significant. Densitometric quantification of protein bands was performed with ImageJ software. Morphometric quantification of M_M_120-1 immunofluorescence images and the colocalization efficiency of M_M_120-1 with other antibodies was carried out with ImageJ interface (NIH, USA) using the particle counter by keeping the threshold value equal for all images. Photoshop CS5 (Adobe, San Jose, USA) was used to prepare the immunofluorescence figures whereas schematics were assembled with CC Illustrator software (Adobe, San Jose, USA).

## Results

### Characterization of p62^-/-^ and p62hep-/- Mice

To study the role of p62 in MDB formation *in vivo*, we generated p62 total (*p62*^*-/-*^) and hepatocyte-specific (*p62hep*^*-/-*^) knockout mice by Cre-loxP technology (S1A Fig). The genomic deletion of p62 was confirmed by genotyping PCR (S1B Fig, S1 Table) and reverse transcriptase PCR (RT-PCR) (S1C Fig). Both *p62*^*-/-*^ and *p62hep*^*-/-*^ and their corresponding littermate controls *p62*^*f/f*^ and *p62hep*^*+/+*^ mice were born at the expected Mendelian ratio and had a normal life span. To induce MDB formation, mice were fed a diet containing 0.1% DDC starting at the age of 2 months for 8 weeks. Among DDC-exposed animals, *p62*^*-/-*^ mice displayed a significantly higher body weight than *p62*^*f/f*^ mice whereas no difference was seen between *p62hep*^*+/+*^ and *p62hep*^*-/-*^ mice (S2A Fig). As reported previously, DDC treatment induced a significant increase in liver-related serum enzymes and liver/body weight ratios [18]. However, these parameters did not differ significantly among genotypes (S2C and S2D Fig).

### p62 Deficiency Prevents the Maturation of MDBs

**Fig 1:**
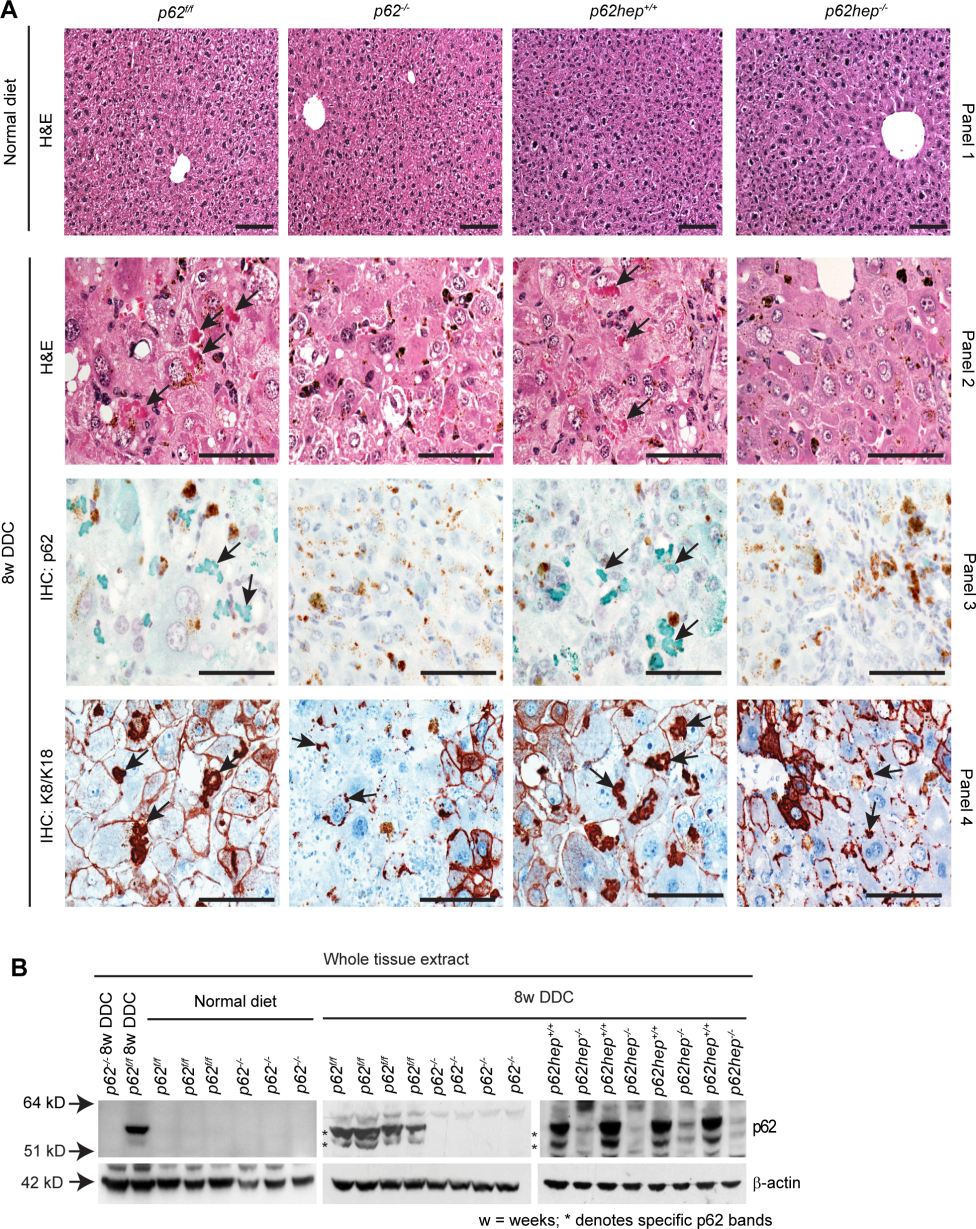
Loss of p62 impairs the formation of DDC-induced MDBs. **(A)** H&E staining was performed on liver sections of *p62*^*f/f*^, *p62*^*-/-*^, *p62hep*^*+/+*^and *p62hep*^*-/-*^ mice fed a normal diet (panel 1) or DDC-containing diet for 8 weeks (panel 2). Immunohistochemistry (IHC) with antibodies against p62 (panel 3) and K8/K18 (panel 4) was performed on DDC-intoxicated livers of the indicated genotypes. No morphological signs of liver injury and no MDBs were found in livers under normal diet (panel 1). Large and distinct MDBs (arrows) were only visible in DDC-fed *p62*^*f/f*^ and *p62hep*^*+/+*^ mice in H&E stained sections (panel 2). These MDBs were also decorated by p62 (green; panel 3) and K8/K18 antibodies (red; panel 4). In *p62*-deficient mice, MDBs are small, less distinct and only decorated by K8/K18 antibodies (arrows; panel 4). MDB-containing hepatocytes display diminished or lacking keratin cytoskeleton (empty cells). Brown pigment in the livers indicates protoporphyrin accumulation. (Scale bar = 50 μm). **(B)** Immunoblotting revealed abundant p62 expression in livers of DDC-fed *p62*^*f/f*^ and *p62hep*^*+/+*^ mice upon DDC-intoxication while no p62 signal was detected in *p62*^*-/-*^ liver. The minimal p62 signal detected in *p62hep*^*-/*-^ livers reflects preserved p62 expression in non-hepatocytic cells. β-actin was used as a loading control.

Histological analysis of livers from *p62*^*f/f*^, *p62*^*-/-*^, *p62hep*^*+/+*^and *p62hep*^*-/-*^ mice fed DDC for 8 weeks (Fig 1A, panel 2), in contrast to animals fed a normal diet (Fig 1A, panel 1), revealed the occurrence of characteristic features of DDC-induced liver injury, such as porphyria (brown pigment deposition), bridging fibrosis, inflammation and ballooning of hepatocytes (compare panels 1 and 2). No differences in these morphological features were found between the different genotypes. Interestingly, the typical dense and distinct eosinophilic ‟classical” MDBs regarded as ‟mature” MDBs were only detected in H&E-stained liver sections of DDC-intoxicated *p62*^*f/f*^ and *p62hep*^*+/+*^ mice (Fig 1A, panel 2) whereas in *p62*-deficient livers, MDBs were smaller, granular and less distinct (Fig 1A, panel 2). Immunohistochemical staining for K8/K18 confirmed the presence of enlarged ‟empty” hepatocytic cells lacking keratin cytoskeleton in all genotypes of DDC-fed mice (Fig 1A, panel 4). Keratin and p62 immunohistochemistry and immunoblotting confirmed the genotypes of the animals as well as the formation of MDBs (Fig 1A, panels 3–4 and Fig 1B).

**Fig 2:**
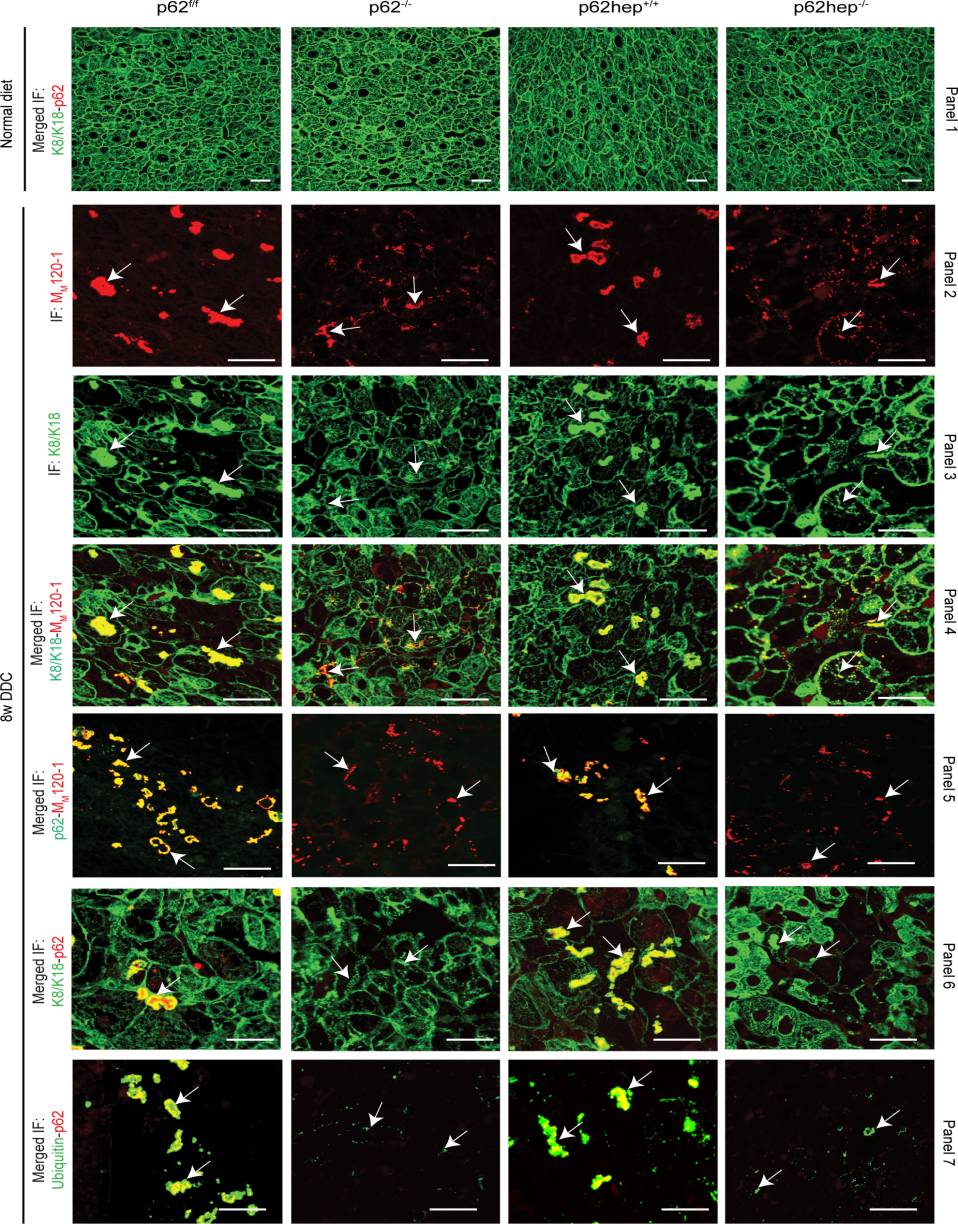
Loss of p62 impairs the formation of large MDBs. Double immunofluorescence staining with K8/K18 (green) and p62 (red) antibodies was performed on liver sections of *p62*^*f/f*^, *p62*^*-/-*^, *p62hep*^*+/+*^and *p62hep*^*-/*^ mice under normal diet (panel 1). To detect MDBs, double immunofluorescence staining with M_M_120-1 (red) and K8/18 antibodies (green) was performed on liver sections of 8 weeks DDC-intoxicated mice (n ≥10). Note that MDBs were found in all genotypes. However, *p62*^*f/f*^ and *p62hep*^*+/+*^ mice developed large aggregates while only small inclusions were observed in *p62*^*-/-*^ and *p62hep*^*-/-*^ mice (panels 2–5) (scale bar = 20μm). To confirm the absence of p62 in MDBs present in p62-deficient livers, various combinations of double immunofluorescence stainings with p62 (green) + M_M_120 (red), K8/K18 (green) + p62 (red) and ubiquitin (green) + p62 (red) antibodies were performed on DDC-treated liver of the different genotypes (panels 5–7. The arrows are used to indicate few MDBs among all the MDBs positive for keratin, M_M_120-1 or p62 to underline the observations (panels 2–7).

**Fig 3:**
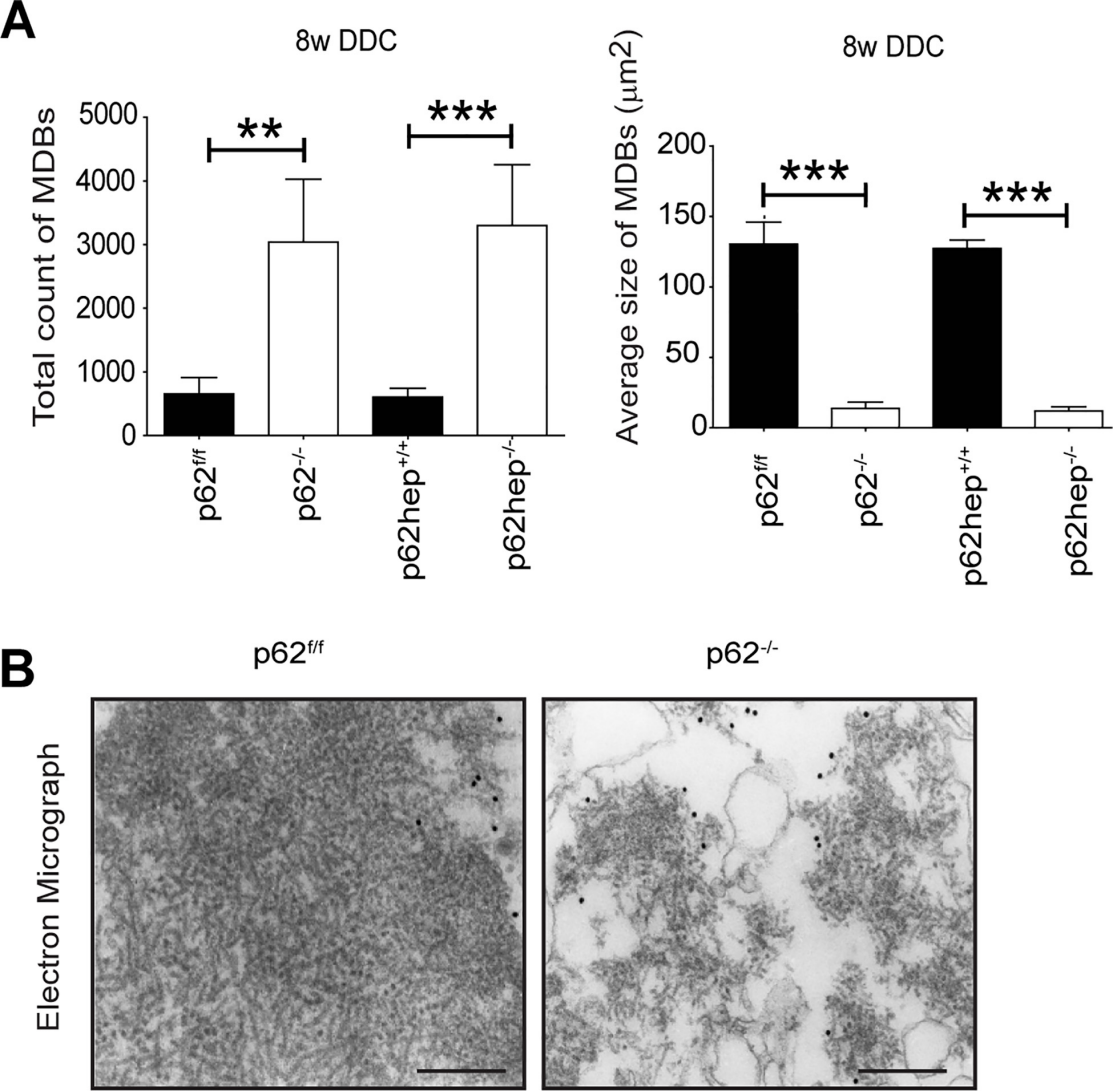
**Loss of p62 influences the size but not the filament ultrastructure of MDBs (A)** Morphometric scoring of the M_M_120-1 immunofluorescence confirmed the visual impression that *p62-*deficient mice contained more small MDBs but rarely developed larger inclusions. (n ≥10) Values are expressed as mean ± SEM. **p<0.01, ***p< 0.001. **(B)** Electron micrographs depict characteristic MDBs with densely but haphazardly arranged filaments in *p62*^*f/f*^ mice while only small filamentous MDBs are seen in *p62*^*-/-*^ mice. Note that the ultrastructure of the MDB filaments is identical in both genotypes. Scale bar = 500 nm.

To further monitor the formation of MDBs, we relied on the detection of M_M_120-1 antigen that constitutes a constant component of MDBs [10, 18]. Double immunofluorescence staining with M_M_120-1 antibody and antibodies to K8/K18 revealed predominantly smaller, granular and less distinct aggregates in DDC-intoxicated *p62*-deficient livers whereas both small granular as well as large MDBs were seen in *p62*^*f/f*^ and *p62hep*^*+/+*^ livers (Fig 2, panels 2–4). The morphometrical analysis confirmed smaller but more numerous aggregates in DDC-intoxicated *p62*-deficient livers than in corresponding *p62*^*f/f*^ and *p62hep*^*+/+*^ livers (Fig 3A). No alterations of the keratin cytoskeleton were observed in livers under a normal diet (Fig 2, panel 1). The absence of p62-positive aggregates in DDC-intoxicated p62-deficient animals was confirmed by performing various combinations of double immunofluorescence stainings with antibodies against MDB-specific components (Fig 2, panels 5–7). Moreover, we observed a rather weak ubiquitin staining of p62-deficient MDBs when compared to the pronounced colocalization of ubiquitin in MDBs of *p62*^*f/f*^ and *p62hep*^*+/+*^ livers (Fig 2, panel 7).

Immunoelectron microscopy with M_M_120-1 antibody revealed that *p62*-deficient livers contained smaller aggregates when compared to larger MDBs in control livers, which however resembled the classical filamentous type II MDBs, occasionally, with electron-dense amorphous material as described for type III MDBs [5] (Fig 3B). Therefore, on the ultrastructural level, no differences existed between the filamentous material of MDBs in p62-deficient and corresponding control animals.

### Loss of p62 Does Not Affect Keratin Modifications but Impairs the Binding of NBR1 and Other Proteins to MDBs

**Fig 4:**
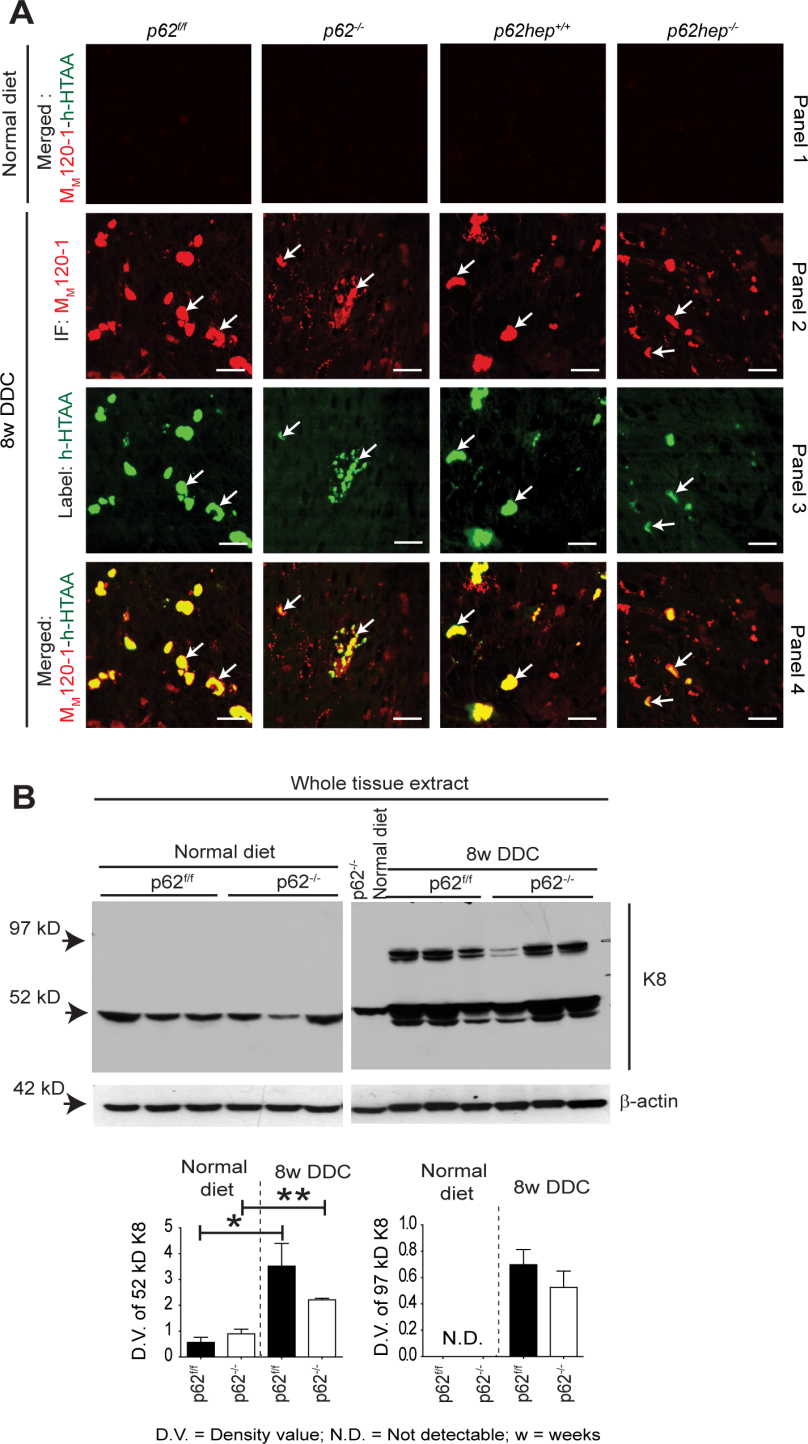
p62 affects neither the extent of DDC-induced protein misfolding nor keratin cross-linking. **(A)** A combination of immunofluorescence staining with M_M_120-1 antibody (red) and labeling with the luminescent conjugated oligothiophene h-HTAA (green) was performed on liver sections of *p62*^*-/-*^, *p62*^*f/f*^, *p62hep*^*-/-*^ and *p62hep*^*+/+*^mice fed a normal diet (panel 1) or DDC for 8 weeks (panels 2–4). Mice fed a normal diet did not show any positive labeling with h-HTAA or M_M_120-1 (panel 1). DDC-intoxicated livers showed colocalization (yellow) of h-HTAA and M_M_120-1 indicating the presence of cross β-sheet conformation in MDBs (arrows) (panel 2; n ≥10) (scale bar = 20μm). **(B)** Whole liver tissue extracts of *p62*^*f/f*^ and *p62*^*-/-*^ mice fed a normal diet or 8weeks DDC were analyzed by western blotting with a K8-specific antibody and densitometric quantification was performed (n = 3). DDC-treatment resulted in an increased amount of K8 (52 kD; bands with lower molecular mass reflect degradation products) and formation of crosslinked K8 dimers (ca 97 kD). However, the extent of dimer formation did not differ significantly between *p62*^*-/-*^ and *p62*^*f/f*^ mice. β-actin was used as a loading control. *p<0.05, **p<0.01.

Keratins are the primary components of MDBs. To analyze molecular structural features of MDBs, we used the luminescent conjugated oligothiophene h-HTAA, which labels cross β-sheet structure of proteins [16]. Double staining with h-HTAA and the M_M_120-1 antibody showed no positivity for cross β-sheet structures in hepatocytes of p62-deficient and corresponding *p62*^*f/f*^ and *p62help*^*+/+*^ control mice fed a normal diet (Fig 4A, panel 1). On the other hand, in DDC-fed mice both p62-deficient and ‟mature” MDBs were positive for cross β-sheet conformation indicating that DDC-induced structural changes of K8 to cross β-sheet conformation in MDBs were independent of p62 (Fig 4A, panel 2–4). The formation of high molecular weight cross-linked K8 species, increased amount of keratin expression (Fig 4B), and altered K8>K18 mRNA ratio **(**S3A Fig) observed after DDC exposure also did not differ among the genotypes. However, the protein levels of transglutaminase 2 (Tg2), which was reported to form glutamine-lysine crosslinks of keratins in MDBs [13], were decreased in DDC-intoxicated *p62*^*-/-*^ livers in comparison to *p62*^*f/f*^ livers (S3B Fig).

**Fig 5:**
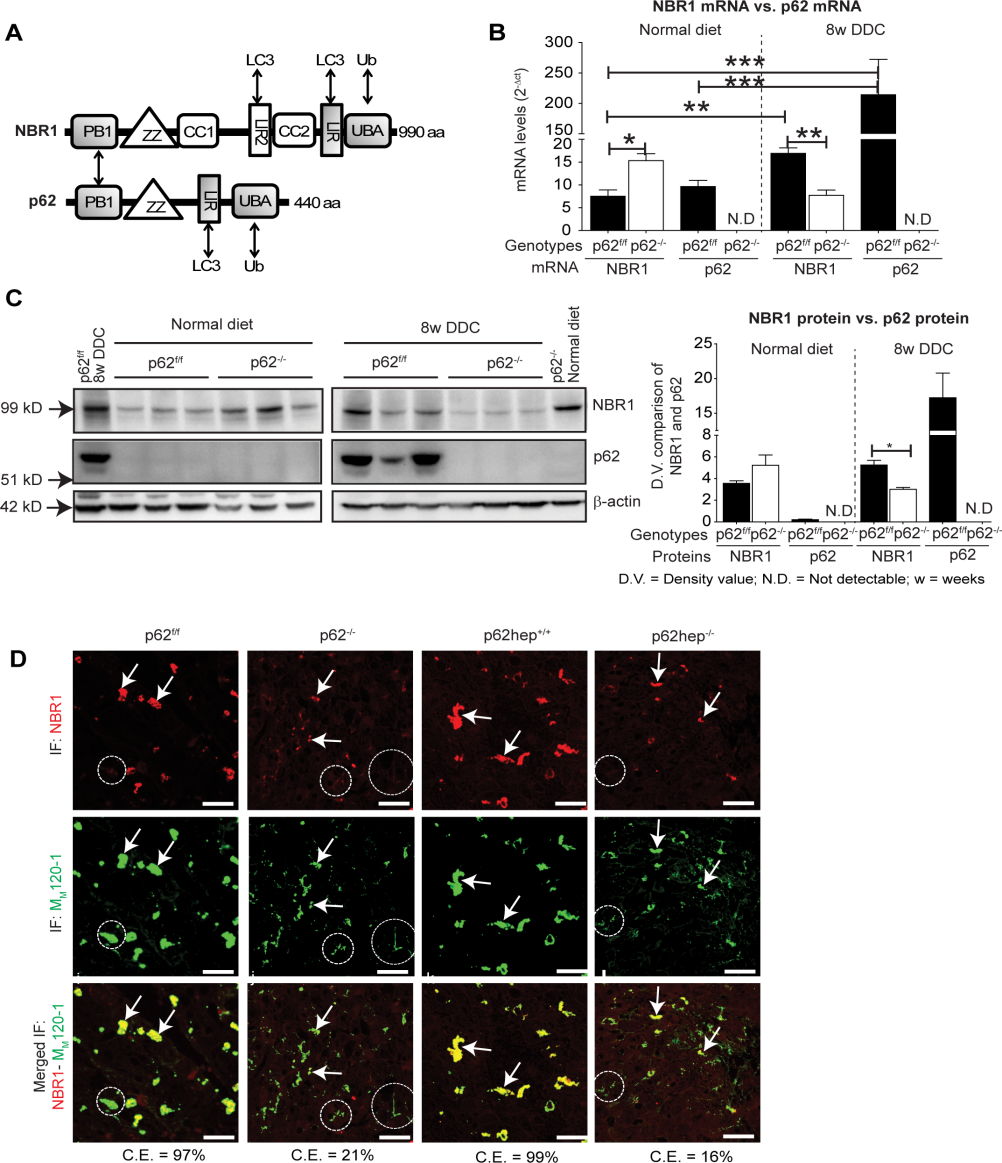
Loss of p62 impairs recruitment of NBR1 to MDBs. **(A).** The scheme (modified from [34]) depicts the protein domain architecture of NBR1 and p62. The reported interaction partners and conserved domains are highlighted by double headed arrows and gray boxes, respectively. PB1, Phox and Bem1p domain; ZZ, zinc-binding domain; CC, coiled-coil domain; LIR, LC3-interacting region; UBA, ubiquitin-associated domain; Ub, ubiquitin. NBR1 and p62 interact via their PB1 domains [34]. **(B)** qPCR analyses of NBR1 and p62 mRNA expression in livers of p62^f/f^ and p62^-/-^ mice under normal diet or 8 weeks DDC. 18S rRNA was used as a normalization control (n = 5). **(C)** Whole liver tissue extracts of p62^f/f^ and p62^-/-^ mice fed a normal diet or 8 weeks DDC were immunoblotted for NBR1 (99 kD) and p62 (~55 kD). A sample from DDC-treated p62^f/f^ liver was run on the same gel with liver samples from mice fed a normal diet (left panel) as common reference (right panel). Densitometric analyses of p62 and NBR1 are also shown (n = 3). *p<0.05, **p<0.01, ***p<0.001**(D)** Double immunofluorescence staining with M_M_120-1 (green) and NBR1 antibodies (red) visualized the distribution of both proteins in livers of mice treated with DDC for 8 weeks. An extensive co-localization between NBR1 and M_M_120-1 (C.E = colocalization efficiency) was seen in animals with intact p62 production (*p62*^*f/f*^ C.E = 97%, and *p62hep*^*+/+*^ C.E = 99%) but not in the total (*p62*^*-/-*^ C.E = 21%) and liver-specific (*p62hep*^*-/-*^ C.E = 16%) *p62*-knockout animals (scale bar = 20μm). NBR1-positive and -negative MDBs are highlighted by arrows and circles, respectively.

Since NBR1 can also be detected in large ‟classical” MDBs and shares both structural and functional similarities with p62 (Fig 5A) [34], we investigated whether NBR1 is able to compensate for the loss of p62 in MDB formation. To this aim, we compared the mRNA and protein levels of p62 and NBR1 in p62^f/f^ and p62^-/-^ livers. Mice fed a normal diet showed similar mRNA levels of NBR1and p62 (Fig 5B). Of note, an increased expression of NBR1 protein was observed in in western blots of total liver extracts of p62-deficient mouse livers when compared to p62^f/f^ livers (Fig 5C, left panel). The minimal levels of p62 seen in the western blot are due to very low concentrations of p62 in mice fed a normal diet (non stressed situation).

DDC-intoxication led to a dramatic increase in both mRNA and protein levels of p62 (Fig 5B). In contrast to p62, there was only a slight increase of NBR1 in DDC-treated p62^f/f^ livers (Fig 5B and 5C, right panel). Surprisingly, NBR1 mRNA and protein expression was markedly reduced in DDC-intoxicated p62-deficient livers as compared to p62-deficient mice fed a normal diet (Fig 5B and 5C). Moreover, double immunofluorescence staining for NBR1 and M_M_120-1 antigen demonstrated an almost perfect overlap of both antigens in DDC-treated p62^f/f^ and p62hep^+/+^ livers whereas a much weaker colocalization was seen in *p62*-deficient livers (Fig 5D, Table 1). Triple staining for M_M_120-1 antigen, NBR1 and ubiquitin revealed that only MDBs containing ubiquitin were also positive for NBR1 (S4 Fig). This demonstrates that p62 enhances the binding of NBR1 to MDBs, but the latter does not compensate for p62’s role in the maturation of MDBs.

**Table 1:** Quantitative assessment of MDB components in p62^f/f^ and p62^-/-^ livers.

	Colocalization efficiency	
MDB components	p62^f/f^	p62^-/-^
M_M_120-1	100%	100%
Keratin 8/18	100%	100%
p62	100%	0%
NBR1	97%	21%
Ubiquitin	96.2%	19.8%
Hsp25	46.7%	0%
SMI 31	100%	0%

Double immunofluorescence staining with antibodies against known MDB components was correlated with the results of M_M_120-1immunostaining, which served as common reference, and colocalization efficiency was quantified for various proteins present in MDBs.

Next, we examined the expression of the chaperones Hsp25 and Hsp70 in DDC-intoxicated *p62*^*f/f*^ and *p62*^*-/-*^ livers. Interestingly, double immunofluorescence staining with antibodies to Hsp25 and the M_M_120-1 antigen (Table 1) revealed that the absence of p62 impaired the binding of Hsp25 to MDBs. Moreover, the protein levels of Hsp25 were reduced in DDC-intoxicated *p62*^*-/-*^ livers in comparison to *p62*^*f/f*^ livers (S3B Fig). On the other hand, Hsp70 protein was not different between DDC-intoxicated *p62*^*f/f*^ and *p62*^*-/-*^ livers (S3B Fig).

In the context of characterizing possible impacts of the absence of p62 on the composition of MDBs we have also investigated the presence of the phosphoepitope SMI 31 in MDBs since it has been described as common feature of MDBs and neurofibrillary tangles (NFTs) [11]. We observed that the SMI 31 epitope was not present in p62-deficient MDBs which demonstrates that the SMI 31 epitope in MDBs is located on p62 and not on tau protein as originally reported for NFTs (Table 1, S3C Fig).

### p62 Provides Stability to MDBs

**Fig 6:**
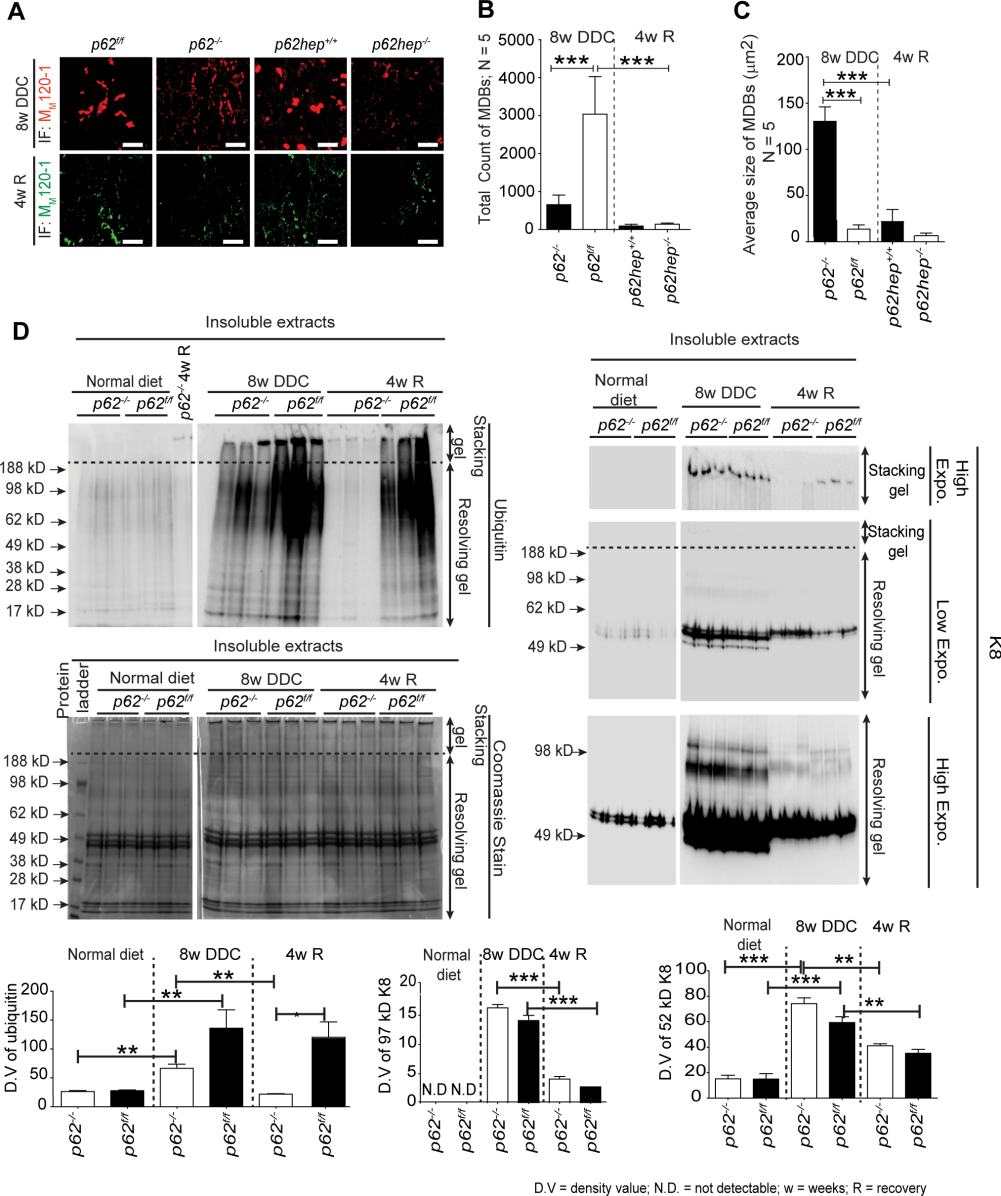
Characterization of MDBs at 4 weeks of recovery after 8 weeks of DDC intoxication. **(A)** Double immunofluorescence staining of liver sections with M_M_120-1 and K8/K18 antibody visualized MDBs in livers of mice fed DDC for 8 weeks (8w DDC) as well as in livers of mice that were subsequently recovered on a standard diet for four weeks (4wR). Recovery led to a substantial reduction of MDBs both in mice with intact p62 production (*p62*^*f/f*^ and *p62hep*^*+/+*^) and total (*p62*^*-/-*^)/liver-specific (*p62hep*^*-/-*^) *p62* knockouts. **(B)** Morphometrical analysis showed that after recovery there was a significant reduction of the number of MDBs in the *p62*-deficient groups (n = 5). **(C)** No significant difference in MDB size was observed after recovery in the different genotypes, which confirmed the observations of immunofluorescence staining (n = 5). **(D)** Immunoblotting of insoluble liver protein extracts with antibodies against keratin 8 (K8) and ubiquitin and corresponding densitometric quantification (n = 5) showed that loss of p62 did not affect the accumulation of insoluble ubiquitinated proteins or formation of cross-linked high molecular mass K8 species during DDC intoxication. However, the disappearance of ubiquitinated proteins and K8 cross-linked proteins during the recovery period was accelerated in *p62*-deficient mice suggesting that the inclusions were less stable. A Coomassie-stained gel for indicated genotypes and treatment shows the protein loading used for western blotting. Values are expressed as mean ± SEM. (n = 5). *p<0.05, ***p< 0.001).

To determine whether p62 alters MDB dynamics, we analyzed the amount of inclusions in mice recovered from DDC intoxication on a standard diet for four weeks. As reported previously [7, 18], the recovery from DDC intoxication resulted in an almost complete disappearance of large MDBs (Fig 6A) while small aggregates occasionally persisted at the cell periphery in proximity to desmosomes (S5 Fig). The numerical reduction of MDBs was much more pronounced in *p62*-deficient than in control livers as confirmed by morphometry (Fig 6B and 6C). In contrast, the reduction in size of MDBs was more prominent in controls (Fig 6C). In addition, insoluble ubiquitinated proteins and the high molecular weight insoluble K8 species were found at similar levels in DDC-fed *p62*^*f/f*^ and *p62*^*-/-*^ mice, but disappeared more quickly in *p62*-deficient animals upon recovery (Fig 6D, see stacking gels). Moreover, no changes in the over-expression of K8 (52 kD and 97 kD) was observed in DDC-treated livers irrespective of the genotype (Fig 6D, see resolving gel). Ubiquitin levels were slightly decreased in DDC-treated livers of p62-deficient mice (Fig 6D, resolving gel).

Further, we measured the levels of p62’s binding partner microtubule-associated light chain 3-II to the light chain 3-I (LC3-II/LC3-I) ratio in the untreated control, DDC-treated and recovered mouse livers (S6 Fig). In chronic DDC intoxication, we observed an increased LC3-II to LC3-I ratio in all genotypes when compared to untreated controls indicating autophagic activity. LC3-II to LC3-I ratios decreased in the recovery period to levels comparable to the untreated controls. There was no significant difference in LC3-II to LC3-I ratios between the genotypes (S6 Fig).

### p62 and the Cellular Defense Mechanisms

**Fig 7:**
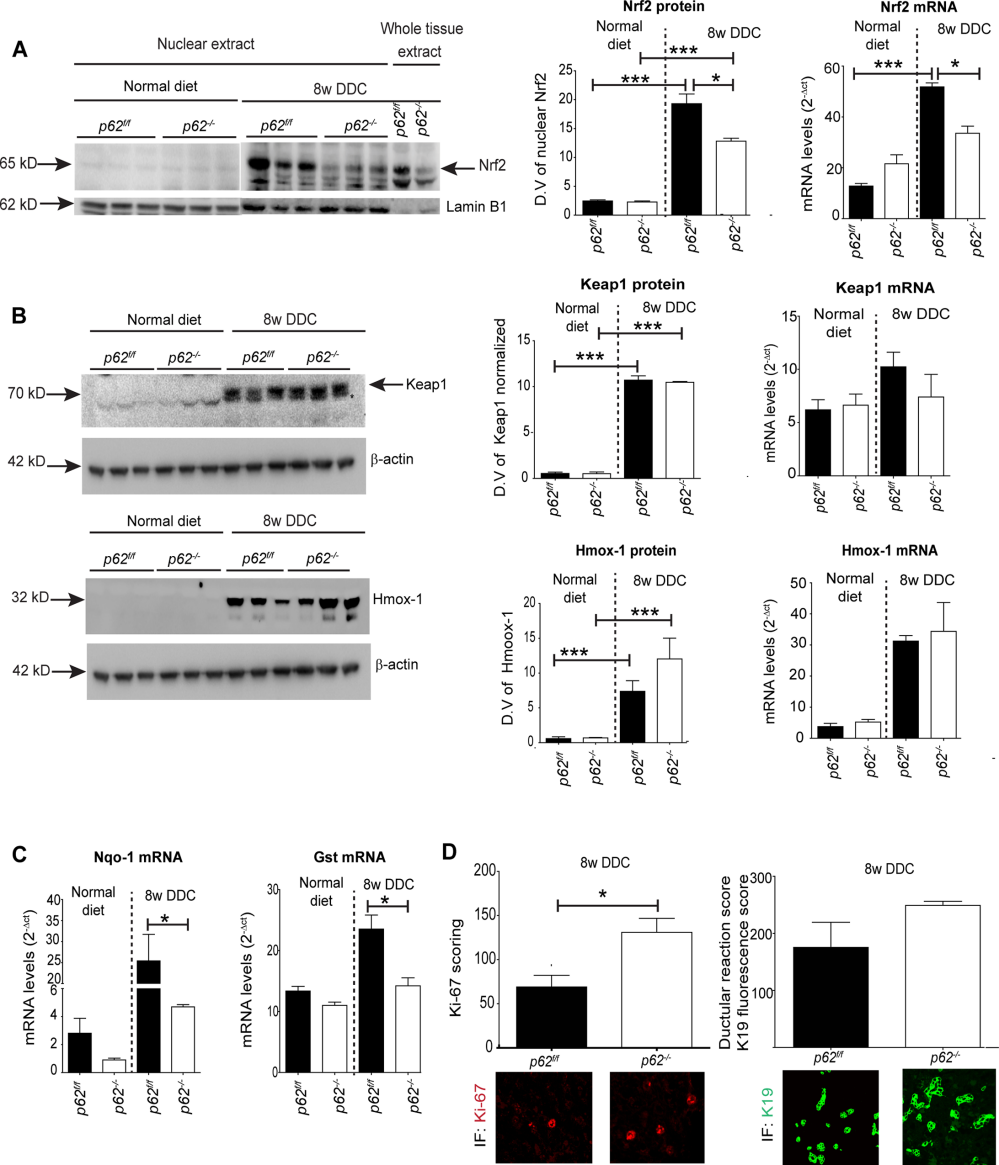
Absence of p62 alters cellular defense mechanisms. **(A)** Liver nuclear extracts and whole tissue extracts of *p62*^*f/f*^ and *p62*^*-/-*^ mice fed a normal diet and of mice DDC-intoxicated for 8 weeks were analyzed by immunoblotting with antibody against Nrf2 (65 kD). Furthermore, quantitative qPCR analyses of Nrf2 mRNA (n = 5) was performed from the same mice. Corresponding densitometric quantification is shown for nuclear extracts (n = 3). Lamin B1 (62 kD) was used as nuclear loading control. **(B)** Liver whole tissue extract from *p62*^*f/f*^ and *p62*^*-/-*^ mice fed a normal diet or 8 weeks DDC were immunoblotted for Keap1 (70 kD) and Hmox1 (32 kD) and qPCR analyses were performed for Keap1 and Hmox-1 mRNA (n = 5). Densitometric quantification for Keap1 and Hmox-1 are shown (n = 3). β-actin was used as a loading control. **(C)** qPCR analyses of Nqo1 and Gst mRNA levels were performed in livers of mice livers (n = 5) fed normal diet or after 8 weeks of DDC-intoxication. 18S rRNA was used as a normalization control. **(D)** Morphometric assessment of Ki-67-positive hepatocytes (Ki-67 score) and K19-positive bililary cells (ductular reaction score) was made by counting at least ten high power fields (400X) of five DDC-intoxicated livers of indicated genotypes stained with Ki-67 and K19 antibodies. At least five animals per genotype were used for qPCR, Ki67 and ductular reaction scoring. Values are expressed as mean ± SEM. **p< 0.01, *p<0.05.

Previous studies with the DDC model suggested that the increased accumulation of p62 and MDB formation is, at least in part, a response of the liver to oxidative stress [35]. Nrf2 (Nuclear factor (erythroid-derived 2)-like 2)-antioxidant pathway is a key signalling pathway determining the cellular defense against oxidative stress [36]. It regulates the expression of cytoprotective genes involved in the detoxication and elimination of reactive oxidants by enhancing cellular antioxidant capacity. Upon DDC intoxication, Nrf2 protein was increased in liver nuclear extracts of p62^f/f^ and p62^-/-^ (Fig 7A) mice suggesting dissociation of Nrf2 from Keap1 complex and nuclear translocation of Nrf2, followed by an increase in transcript levels of Nrf2 target genes, such as Nqo1 and Gst (Fig 7C). Interestingly, in addition to the translocation of Nrf2 into the nucleus there was also a marked overexpression of the Nrf2 mRNA in DDC-intoxicated livers (Fig 7A) suggesting additional transcriptional mechanisms regulating Nrf2 expression level, which to some extent were influenced by p62 since Nrf2 mRNA expression and the nuclear translocation were slightly reduced in p62-deficient mice when compared to the p62^f/f^ livers. This reduced Nrf2 expression was accompanied by a reduction in Nqo1 and Gst mRNA levels in p62-deficient livers when compared to p62^f/f^ livers (Fig 7C). No significant difference in the transcript and protein levels of Nrf2-interacting partners Keap1 (Kelch-like ECH-associated protein 1) and Nrf2 target gene *Hmox-1* (hemeoxygenase 1) was observed between DDC-intoxicated *p62*^*f/f*^ and *p62*^*-/-*^ livers (Fig 7B). Taken together, these data indicate an impaired cellular defense mechanism in DDC-intoxicated p62-deficient liver leading to more pronounced injury. Although, this was not readily apparent at light microscopy (Fig 1A), the significant increase in the number of Ki-67-positive mitotic cells and the slightly enhanced ductular reaction in *p62*-deficient livers support this assumption (Fig 7D).

## Discussion

MDBs developed in DDC-intoxicated *p62*-deficient mice were smaller, granular and less distinct in their outlines when compared to that of the control mice (Fig 2). Thus, MDBs in *p62*-deficient livers resembled early stages of MDB development suggesting that the coalescence to larger aggregates is impaired. However, these ‟early” p62-deficient MDBs already displayed the principal features of classical MDBs, i.e., cross β-sheet conformation, type II filamentous ultrastructure and M_M_120-1 positivity (Figs 2–4) [6, 10, 16]. Considering p62’s ubiquitin binding nature, the fact that ubiquitinated-keratin-rich ‟early” MDBs were formed in p62-deficient animals was surprising and suggestive of the presence of other ubiquitin adaptors such as NBR1. However, there were only a few NBR1-positive MDBs in *p62*-deficient livers, the majority of which lacked NBR1 (Fig 4B, Table 1), suggesting that MDB formation is initiated by keratin aggregation independent of p62 or NBR1. This is also supported by previous immunofluorescence microscopic observations [18] and recent proximity ligation studies demonstrating the occurrence of MDBs devoid of p62 [37]. We noticed a (compensatory) increase in the protein and mRNA levels of NBR1 in livers of p62-deficient mice fed a normal diet. However, this increase was reversed upon DDC-intoxication, with significant decrease of NBR1 in the absence of p62 (Fig 5). This suggests a transcriptional and post-transcriptional regulation of NBR1 by p62 during chronic stress. This role of p62 can be extended to other protein aggregation disease models such as α-synucleinopathy, where interdependency of p62 and NBR1 expressions in aggregate formation process was reported [38].

During chronic stress, small heat shock proteins, such as Hsp25 in mouse or Hsp27 in humans act as molecular chaperones by keeping the newly formed misfolded proteins in a folding-competent state until the physiological situation is improved allowing ATP-dependent chaperones like Hsp70 to refold misfolded proteins [32, 39]. The presence of Hsps in protein aggregates implies a futile attempt to prevent protein misfolding and aggregation [39]. Ubiquitinated misfolded aggregates with attached chaperones are then destined to bind cargo adaptor proteins, such as p62 or NBR1 via UBA domain for removal by autophagy [40]. Molecular chaperones such as Hsp25 and Hsp70 were previously described as component of MDBs [41]. In a DDC-primed mouse model, heat shock led to the expression of both Hsp25 and Hsp70 before MDB formation suggesting a functional role of Hsps in MDB pathogenesis [42]. In our study, MDBs in p62-deficient livers were negative for Hsp25 (Table 1, S3 Fig) suggesting a novel cooperative mechanism between p62 and Hsp25 in response to aggregates of misfolded proteins, which needs to be further characterized.

In contrast to the lack of involvement of p62 in the initiation of MDB formation, p62 stabilizes MDBs against degradation as indicated by the pronounced reduction of MDBs and the rapid disappearance of high molecular mass K8 and ubiquitinated proteins in *p62*^*-/-*^ livers in the recovery phase after DDC intoxication (Fig 6). This marked decrease of high molecular weight crosslinked proteins could also be attributed to the observed decrease in Tg2 levels in p62-deficient livers (S3 Fig) [13]. According to earlier reports, stimulated autophagy counteracts MDB formation [27]. However, whether p62 modulates autophagy for the clearance of MDBs after recovery from DDC intoxication is still unclear. In our study, the presence or absence of p62 had no effect on the alterations of LC3-II/ LC3-I ratio in the different treatment groups (S6 Fig). Thus, it is unlikely that p62-dependent impairment of autophagy is responsible for reduced stability of MDBs in p62-deficient mice.

We found no major difference in liver morphology or liver-damage-related serum enzyme levels between DDC-intoxicated control and *p62*-deficient mice, despite differences in MDB formation (S2 Fig, Fig 1). This was surprising since p62 exerts important functions in the cell by affecting NFkB signaling, regulating apoptosis through caspase 8 aggregation and modulating oxidative stress signaling and defense mechanisms [43, 44]. It was previously reported that p62 transcription is regulated by Nrf2 during chronic liver inflammation and oxidative stress [45]. This is particularly pertinent in our model since oxidative stress is considered to play an important role in steatohepatitis of alcoholic and non-alcoholic etiology [46]. In the non-stressed situation, Nrf2 is sequestered in the cytoplasm by binding to its interaction partner Keap1, followed by ubiquitination and proteasomal degradation by E3 ubiquitin ligase Cul3 [47]. Oxidative stress leads to a conformation change in keap1-Cul3 association, inhibiting the enzymatic activity of Cul3, leading to Nrf2 dissociation. Free Nrf2 translocates to the nucleus and initiates the transcription of various antioxidant genes, such as Nqo1 and Gst [48, 49]. Alternately, p62 competitively inhibits the Keap1-Nrf2 interaction, leading to the stabilization of Nrf2 allowing nuclear translocation and expression of cytoprotective genes [43]. DDC intoxication led to nuclear translocation of Nrf2 that was less pronounced in p62-deficient livers than in p62^f/f^ controls, which, at least in part, can be attributed to the known function of p62 as competitive inhibitor of Keap1 [50]. Interestingly, DDC intoxication led to marked upregulation of Nrf2 mRNA level. This effect was weaker in p62 deficiency when compared to p62^f/f^ livers. The decrease in transcript and protein levels of Nrf2 in p62-deficient mice upon DDC-stress can be attributed to the role of p62 as an endogenous modulator of Nrf2 expression during chronic stress situations (Fig 7). This is also mirrored by a further reduction in Nqo1 and Gst mRNA in p62-deficient livers (Fig 7). Similar observations were recently made in a mouse model of hepatocellular carcinoma where chronic stress (Diethylnitrosamine treatment) led to reduced expression of Nrf2 target genes including Gst and Nqo1 mRNA in p62-deficient livers [51]. The impaired activation of proteins involved in cellular defense mechanisms in p62 deficiency could also explain the increased cell turnover as demonstrated by increased Ki-67 positive mitotic cells and slightly enhanced ductular reaction suggesting more pronounced liver injury (Fig 7). These data further strengthen the purported role of p62 in activation of Nrf2 and promoting cell survival by creating a positive feedback loop resulting in enhanced expression of cytoprotective genes.

**Fig 8:**
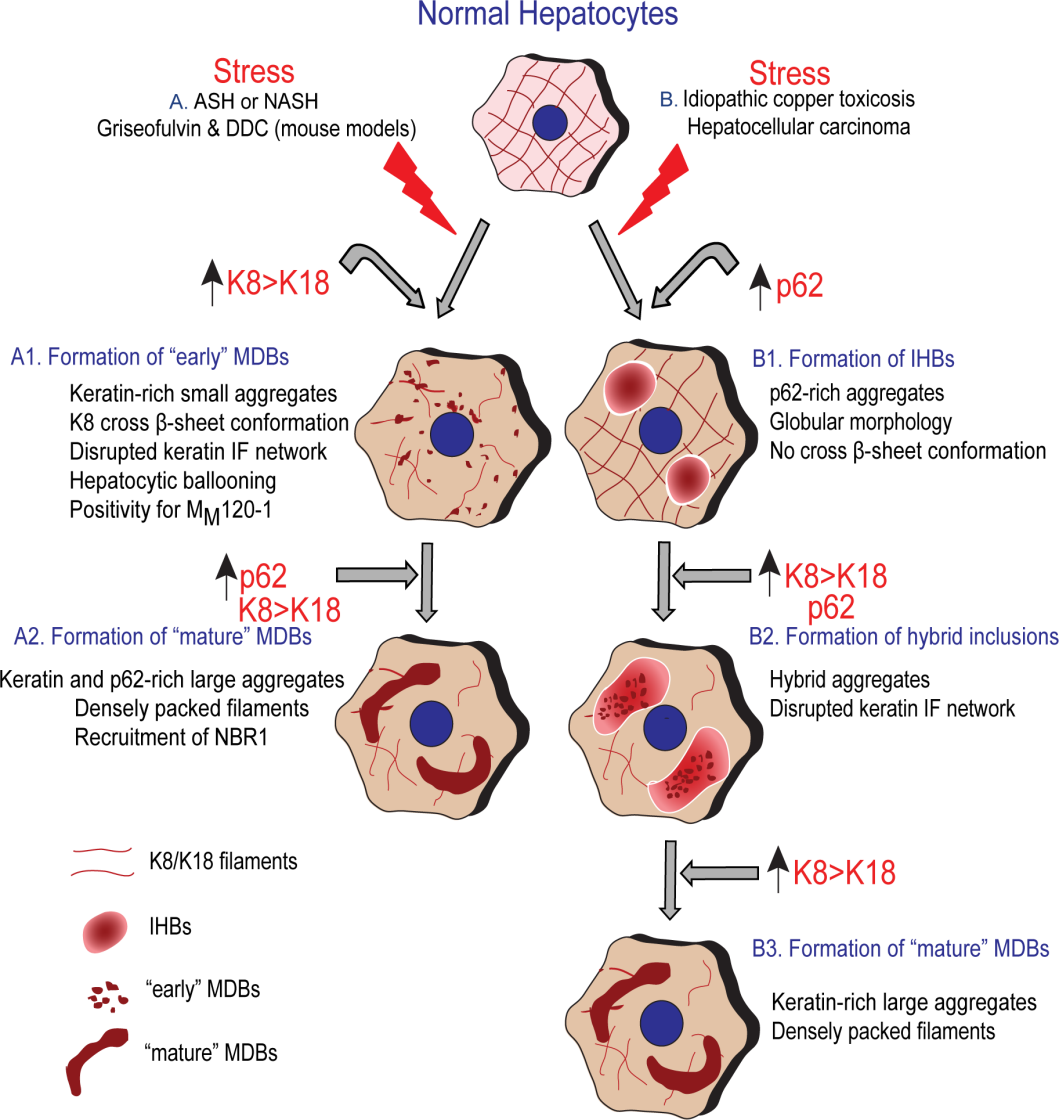
Schematic drawing of the different role of p62 and keratin in two distinct hypothetical pathways of MDB formation. **(A1)** In the first pathway alcohol abuse or metabolic alterations associated with obesity in the context of human alcoholic or non-alcoholic steatohepatitis (ASH or NASH), and DDC or griseofulvin administration in mice trigger the upregulation of keratins (K8 and K18) with, increased K8:K18 ratio and cross β-sheet conformation of K8, which leads to the formation of small ‟early” MDBs with filamentous ultrastructure and M_M_120-1 antigen positivity. **(A2)** The coalescence of small MDBs to form large, compact ‟mature” MDBs occurs via incorporation of p62. Binding of p62 to MDBs also promotes the recruitment of NBR1 and other proteins to MDBs. **(B1)** In the second pathway (found in hepatocellular carcinoma and idiopathic copper toxicosis) p62-containing IHBs, which are negative for keratin and do not show cross β-sheet conformation, may progress to **(B2)** hybrid inclusions (showing mixed features of both IHBs and MDBs) by incorporation of keratins (K8 and K18). **(B3)** The hybrid inclusions may transform to ‟mature” MDBs upon further incorporation of K8 and K18.

On the basis of morphology, ultrastructural and chemical composition, MDBs are related to, but not identical with, IHBs [22]. IHBs can be found in a few cases of HCC [22] and livers of infants with copper toxicosis [23] occasionally in coexistence with MDBs [16, 22]. IHBs contain p62 and ubiquitin (although not constantly) but differ from MDBs by lacking keratins [6]. IHBs can thus be regarded as *in vivo* equivalents of ‟p62 bodies” found in stressed tissue culture cells caused by increased expression of p62 [19, 24, 34]. It can be speculated that p62 bodies can serve distinct cellular functions [21]. For example, p62 may reversibly recruit specific target proteins from their functionally relevant site(s) in the cell. By doing that, the proteins may be stored until the appropriate signal causes a conformational change leading to their release [20]. Our data from this study and previous reports [22] suggest that MDB formation may occur via two different pathways (Fig 8). In the first, which seems to be typical for human steatohepatitis and related animal models (e.g., DDC intoxication), small keratin-containing ‟early” MDB granules are generated in response to stress-induced conformational changes and ubiquitination of keratin; the subsequent incorporation of p62 and NBR1 results in the formation of large ‟mature” MDBs. In the second pathway, which can be proposed on the basis of previous histological and ultrastructural analyses of IHBs in HCCs and idiopathic copper toxicosis, overexpression and accumulation of p62 precede keratin aggregation [5, 51]. The later incorporation of ‟abnormal” keratins into a matrix of aggregated p62 causes the transformation of p62-containing IHBs/aggregates into classical MDBs [5, 22].

## Supporting Information

S1 Fig Generation of total (p62^-/-^) and hepatocyte-specific (p62hep^-/-^) p62-knockout mice. **(A)** The scheme depicts the non-transgenic (*p62*^*NT*^) allele (upper diagram) consisting of 8 exons (numbered rectangles) and the targeting construct consisting of neomycin resistance selection cassette (NeoR), herpes simplex virus thymidine kinase gene cassette (HSV-TK) and three loxP sequences (represented as block triangles). Two of the loxP sites flank NeoR and the third is located between exon 4 and 5 of the *p62* gene. The *p62NeoR*^*flox*^ allele was generated by homologous recombination in ES cells. A deletion of NeoR and the first loxP site resulted in the conditional *p62*^*f*^ allele while an additional ubiquitous excision of exon 1–4 via crossbreeding with Meu-CRE40 mice yielded the constitutive *p62*^*∆ex1-4*^ or *p62*^*-/-*^ knockout. The hepatocyte-specific deletion of exon 1–4 (*p62hep*^*-/-*^) was achieved by breeding *p62*^*f*^ mice with animals expressing Cre-recombinase under the control of the liver-specific albumin promoter (Alb-Cre mice). The schemes also include the localization of the genotyping primers LoxPa/b and CreA/B. **(B)** PCR genotyping of offspring using tail biopsies obtained from the breeding of *p62NeoR*^*flox*^ mice with Meu-Cre40 (left panel) and *p62*^*f/f*^ mice with Alb-Cre mice (right panel). The alleles and their sizes are shown on the right side of the images. **(C)** RT-PCR detects the expression levels of p62 in livers and kidney of the highlighted phenotypes. Weak p62 band in the livers of *p62hep*^*-/-*^ mice likely corresponds to the signal from non-epithelial cells. β-actin was used as reference for normalization.
(TIF)

S2 Fig The extent of DDC-induced liver injury is not affected by loss of p62. **(A)** Body weight, **(B)** liver-to-body weight ratio, **(C)** serum alanine aminotransferase (ALT) **(D)** alkaline phosphatase (AP) levels were measured in 8 weeks DDC-fed 4 months old total (*p62*^*-/-*^*)* and hepatocyte-specific (*p62hep*^*-/-*^) *p62*-knockout mice as well as in their littermates with unaffected hepatic p62 expression (*p62*^*f/f*^ and *p62hep*^*+/+*^). Among DDC-fed mice, *p62*^*-/-*^ mice displayed significantly higher body weight than *p62*^*f/f*^ animals. DDC induced a substantial increase in liver size and liver enzymes. However, none of these parameters differed between *p62*-deficient animals and their respective controls. Values are expressed as mean ± SEM. n = 5, *p<0.05.
(TIF)

S3 Fig Characterization of MDB components. (A) qPCR for K8 and K18 was performed on untreated and DDC-treated livers of p62^f/f^ and p62^-/-^ animals. β-actin was used as reference for normalization. The transcript levels of both K8 and K18 were upregulated in DDC-intoxicated livers of *p62*^*f/f*^ and *p62*^*-/-*^ mice when compared with untreated mice of both genotypes. Moreover, the increase in transcript expression ratio of K8/K18 was observed in both DDC-intoxicated p62-deficient and wildtype mice when compared with untreated controls. (B) Whole tissue extract from 8 weeks DDC-treated livers of *p62*^*f/f*^ and *p62*^*-/-*^ mice were immunoblotted for MDB components Hsp70, Hsp25 and Tg2. The expression of Hsp70 did not differ between *p62*^*f/f*^ and *p62*^*-/-*^ livers. However, the expression of Hsp25 and Tg2 was markedly decreased in *p62*^*-/-*^ livers when compared to wildtypes (C) Double immunofluorescence staining with M_M_120-1 (green) and SMI-31 (red) antibodies was performed on liver sections of DDC-intoxicated p62^f/f^ and p62^-/-^ mice. SMI-31 colocalized with M_M_120-1 in p62^f/f^ livers wheras no colocalization of SMI-31 was observed in p62-deficient MDBs.
(TIF)

S4 Fig Loss of p62 impairs recruitment of NBR1 and ubiquitin to MDBs. Triple immunofluorescence staining with antibodies against M_M_120-1 (MDB marker; blue), NBR1 (green) and ubiquitin (red) visualized the distribution of the respective antigens in *p62*^*f/f*^ and *p62*^*-/-*^ livers intoxicated with DDC for 8 weeks. NBR1-positive and -negative MDBs are highlighted by arrows and dotted circles, respectively. An extensive co-localization of NBR1 and the MDB markers M_M_120-1 and ubiquitin was seen in animals with intact p62 production (*p62*^*f/f*^ and *p62hep*^*+/+*^) but less in the complete (*p62*^*-/-*^) and liver-specific (*p62hep*^*-/-*^) *p62*-knockouts. (Scale bar = 20μm).
(TIF)

S5 Fig Association of MDBs with desmosomes. Double immunofluorescence staining with antibodies against M_M_120-1 (MDB marker; red) and desmoplakin (desmosomal marker; green) visualized the distribution of the antigens in 8 weeks DDC-intoxicated (8w DDC) and 4 weeks recovered (4w R) *p62*^*f/f*^ and *p62*^*-/-*^ mouse livers. MDBs are indicated by arrows. (Scale bar = 20 μm; inset showing higher magnification; scale bar = 10 μm).
(TIF)

S6 Fig Analysis of autophagy-related LC3-II/I ratio in DDC-intoxicated and -recovered livers. Whole tissue extracts from untreated mice, mice fed with DDC for 8 weeks (8w DDC) and from mice recovered on standard diet for four weeks (4w R) after DDC exposure were analyzed by western blotting with an antibody against LC3 as a marker of autophagy activation. Densitometric analysis depicted the intensity of both LC3-I and -II isoforms (dark/light bar) normalized to β-actin. DDC-intoxicated mice showed. higher LC3-II/I ratio but recovered mice acquired attenuation of autophagy (i.e. lower LC3-II/LC3-I ratio). The extent of autophagy did not differ between total *p62*^*-/-*^ and *p62*^*f/f*^ mice of the same treatment regimen.
(TIF)

S1 Table Genotyping PCR. PCR A/B was performed to distinguish between p62 non-transgenic (p62NT), p62 floxed (p62f) and p62 ∆exon1-4 (p62- or p62hep-) while PCR C was performed to detect the presence of Cre-recombinase.
(PDF)

S2 Table List of primers used for genotyping, qPCR and RT-PCR. (PDF)

S3 Table List of Antibodies used for immunofluorescence, immunohistochemistry and western blot. (PDF)
