# Supplementary figures and images for: p62/Sequestosome-1 Is Indispensable for Maturation and Stabilization of Mallory-Denk Bodies
Source: PLoS One. 2016 Aug 15;11(8):e0161083. doi: 10.1371/journal.pone.0161083 (PMC4985067; doi:10.1371/journal.pone.0161083)

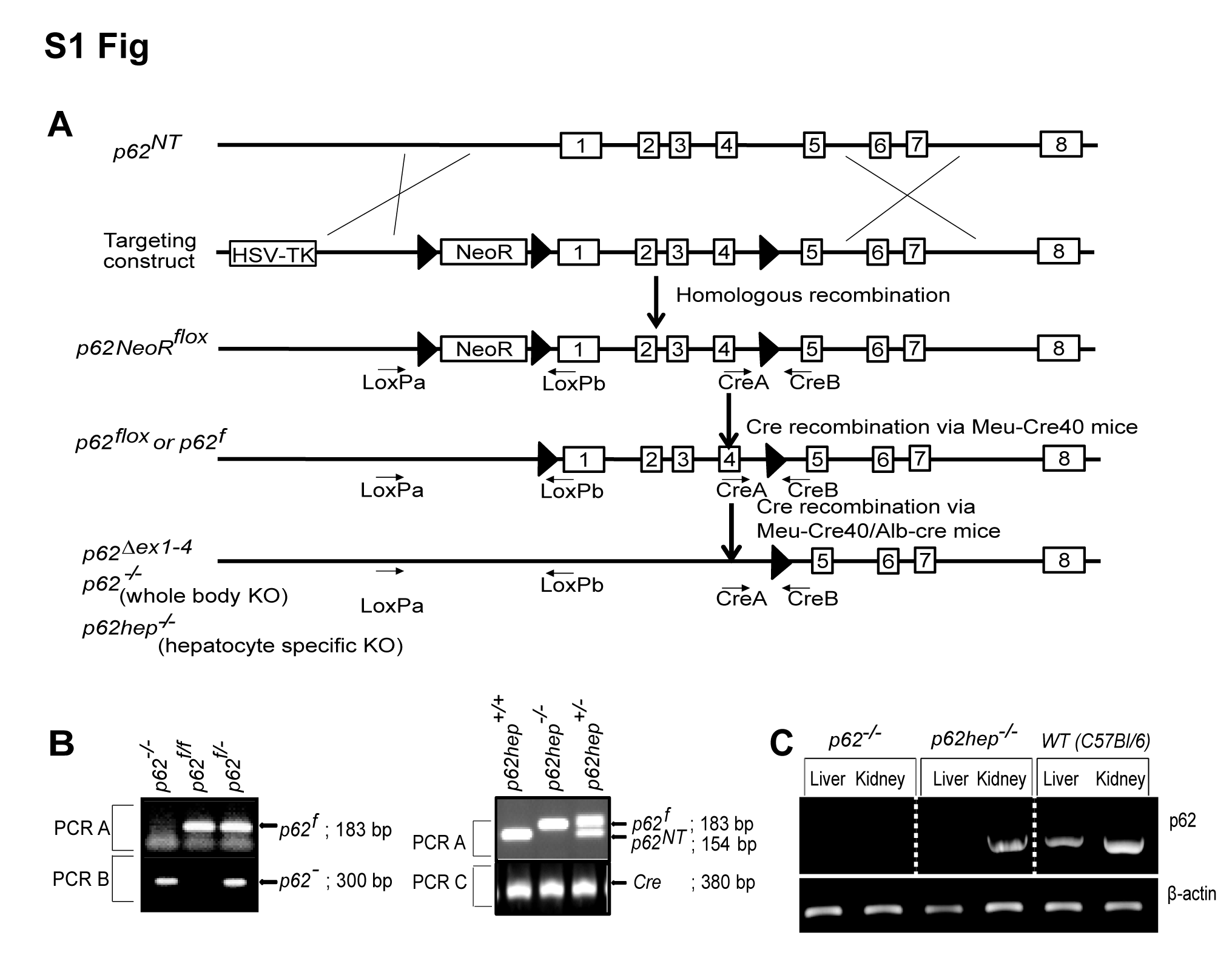

Supplement: S1 Fig — (A) The scheme depicts the non-transgenic (p62NT) allele (upper diagram) consisting of 8 exons (numbered rectangles) and the targeting construct consisting of neomycin resistance selection cassette (NeoR), herpes simplex virus thymidine kinase gene cassette (HSV-TK) and three loxP sequences (represented as block triangles). Two of the loxP sites flank NeoR and the third is located between exon 4 and 5 of the p62 gene. The p62NeoRflox allele was generated by homologous recombination in ES cells. A deletion of NeoR and the first loxP site resulted in the conditional p62f allele while an additional ubiquitous excision of exon 1–4 via crossbreeding with Meu-CRE40 mice yielded the constitutive p62∆ex1-4 or p62-/- knockout. The hepatocyte-specific deletion of exon 1–4 (p62hep-/-) was achieved by breeding p62f mice with animals expressing Cre-recombinase under the control of the liver-specific albumin promoter (Alb-Cre mice). The schemes also include the localization of the genotyping primers LoxPa/b and CreA/B. (B) PCR genotyping of offspring using tail biopsies obtained from the breeding of p62NeoRflox mice with Meu-Cre40 (left panel) and p62f/f mice with Alb-Cre mice (right panel). The alleles and their sizes are shown on the right side of the images. (C) RT-PCR detects the expression levels of p62 in livers and kidney of the highlighted phenotypes. Weak p62 band in the livers of p62hep-/- mice likely corresponds to the signal from non-epithelial cells. β-actin was used as reference for normalization. (TIF) [file pone.0161083.s001.tif]

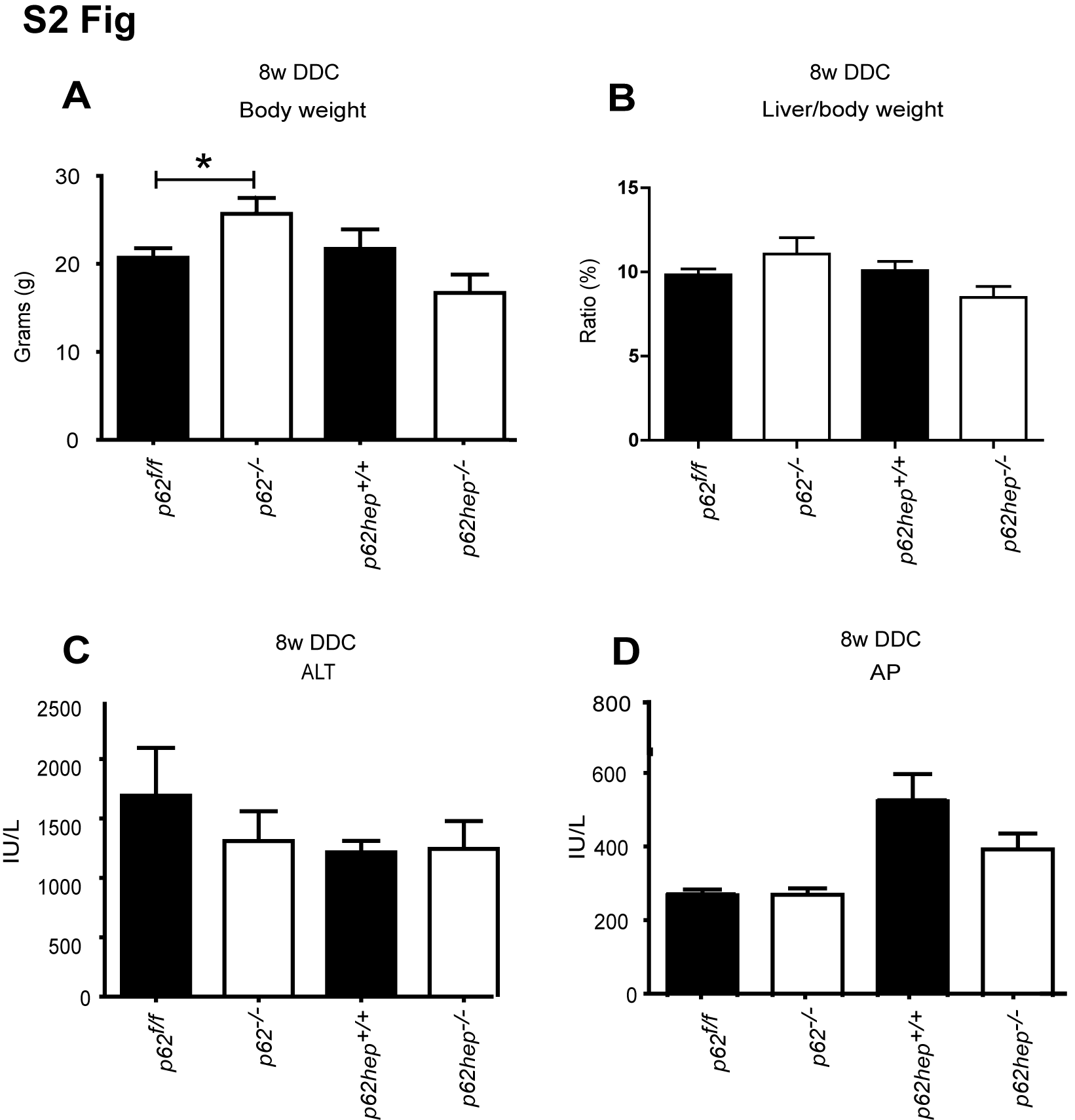

Supplement: S2 Fig — (A) Body weight, (B) liver-to-body weight ratio, (C) serum alanine aminotransferase (ALT) (D) alkaline phosphatase (AP) levels were measured in 8 weeks DDC-fed 4 months old total (p62-/-) and hepatocyte-specific (p62hep-/-) p62-knockout mice as well as in their littermates with unaffected hepatic p62 expression (p62f/f and p62hep+/+). Among DDC-fed mice, p62-/- mice displayed significantly higher body weight than p62f/f animals. DDC induced a substantial increase in liver size and liver enzymes. However, none of these parameters differed between p62-deficient animals and their respective controls. Values are expressed as mean ± SEM. n = 5, *p<0.05. (TIF) [file pone.0161083.s002.tif]

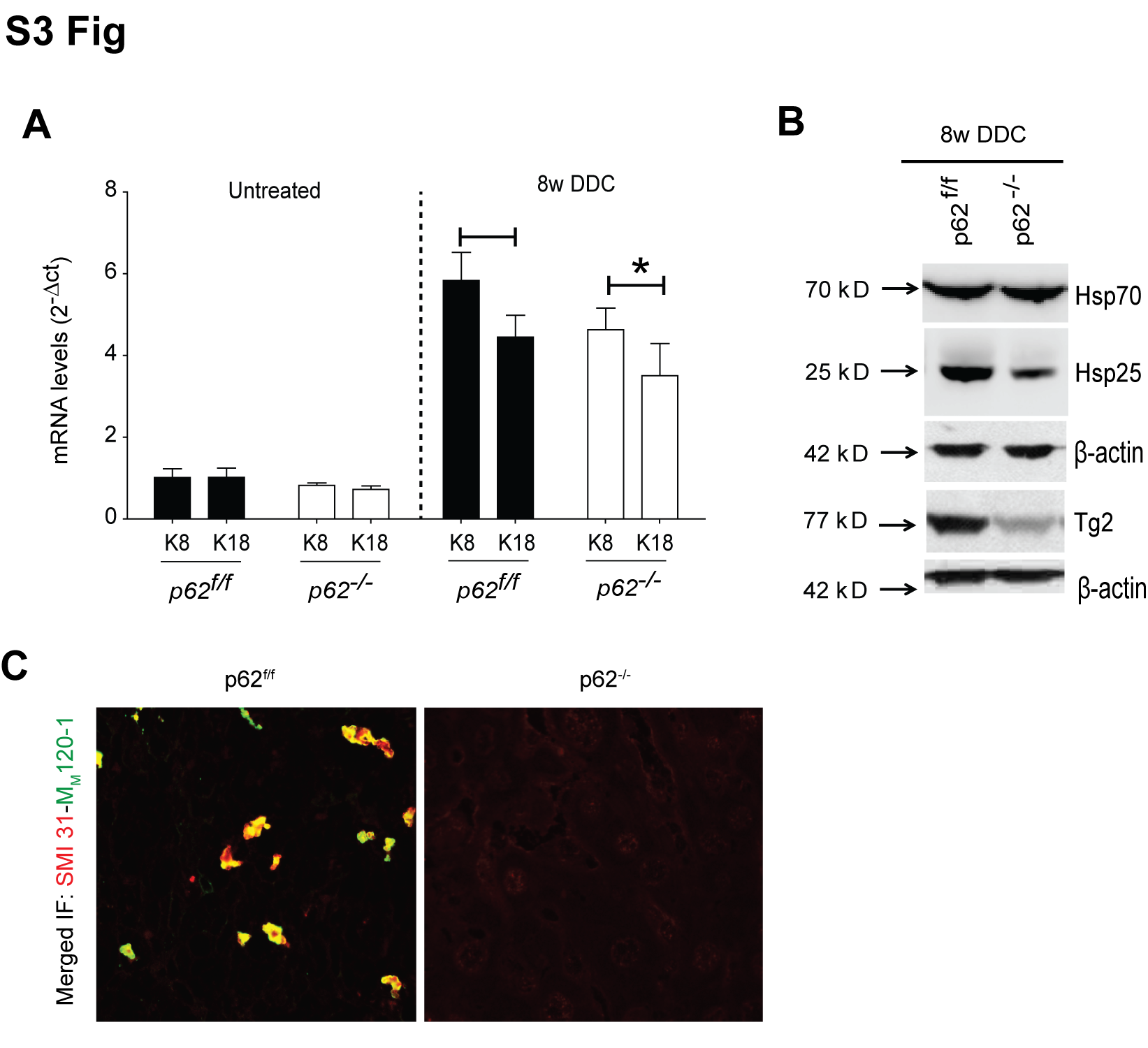

Supplement: S3 Fig — (A) qPCR for K8 and K18 was performed on untreated and DDC-treated livers of p62f/f and p62-/- animals. β-actin was used as reference for normalization. The transcript levels of both K8 and K18 were upregulated in DDC-intoxicated livers of p62f/f and p62-/- mice when compared with untreated mice of both genotypes. Moreover, the increase in transcript expression ratio of K8/K18 was observed in both DDC-intoxicated p62-deficient and wildtype mice when compared with untreated controls. (B) Whole tissue extract from 8 weeks DDC-treated livers of p62f/f and p62-/- mice were immunoblotted for MDB components Hsp70, Hsp25 and Tg2. The expression of Hsp70 did not differ between p62f/f and p62-/- livers. However, the expression of Hsp25 and Tg2 was markedly decreased in p62-/- livers when compared to wildtypes (C) Double immunofluorescence staining with MM120-1 (green) and SMI-31 (red) antibodies was performed on liver sections of DDC-intoxicated p62f/f and p62-/- mice. SMI-31 colocalized with MM120-1 in p62f/f livers wheras no colocalization of SMI-31 was observed in p62-deficient MDBs. (TIF) [file pone.0161083.s003.tif]

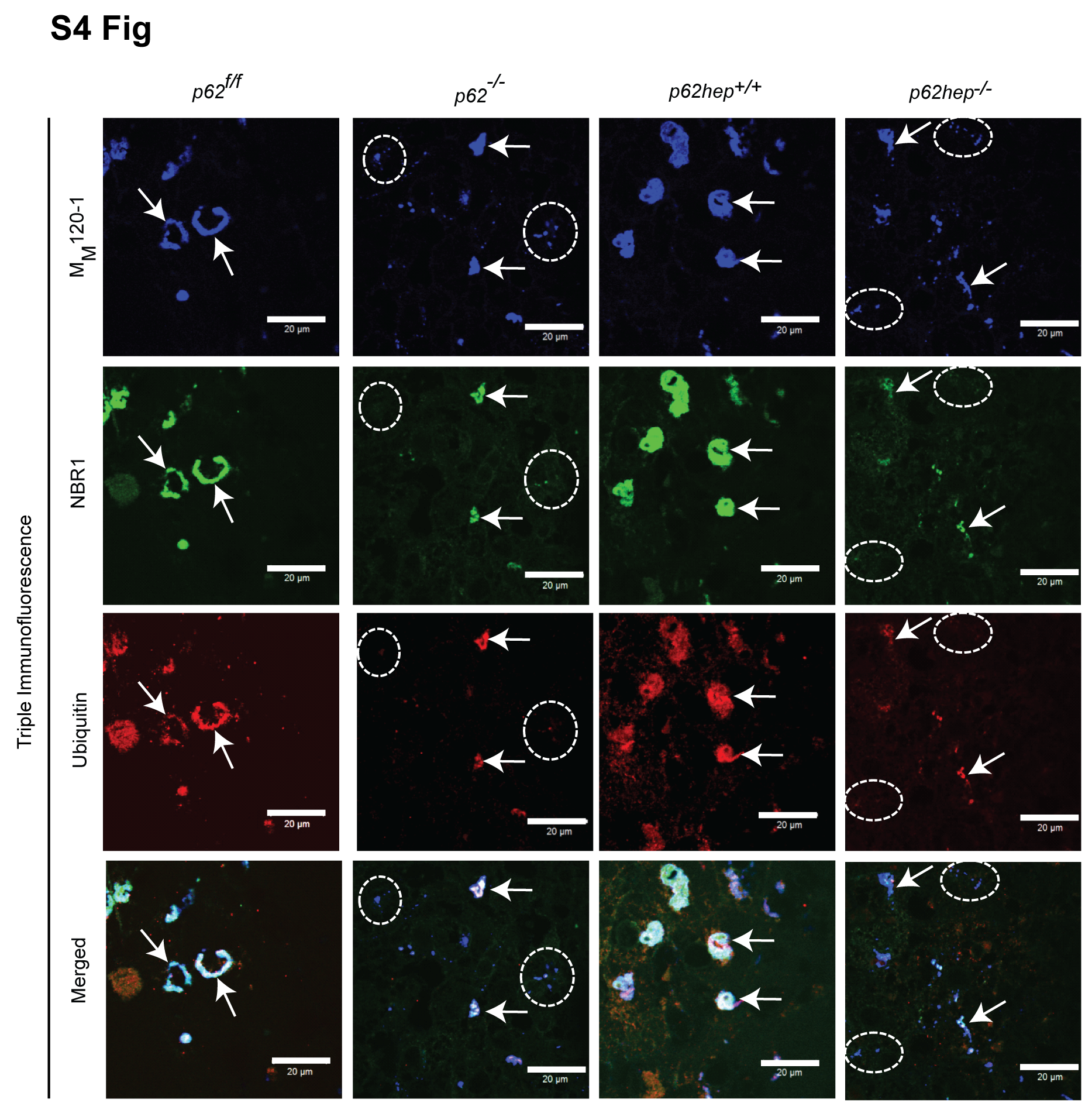

Supplement: S4 Fig — Triple immunofluorescence staining with antibodies against MM120-1 (MDB marker; blue), NBR1 (green) and ubiquitin (red) visualized the distribution of the respective antigens in p62f/f and p62-/- livers intoxicated with DDC for 8 weeks. NBR1-positive and -negative MDBs are highlighted by arrows and dotted circles, respectively. An extensive co-localization of NBR1 and the MDB markers MM120-1 and ubiquitin was seen in animals with intact p62 production (p62f/f and p62hep+/+) but less in the complete (p62-/-) and liver-specific (p62hep-/-) p62-knockouts. (Scale bar = 20μm). (TIF) [file pone.0161083.s004.tif]

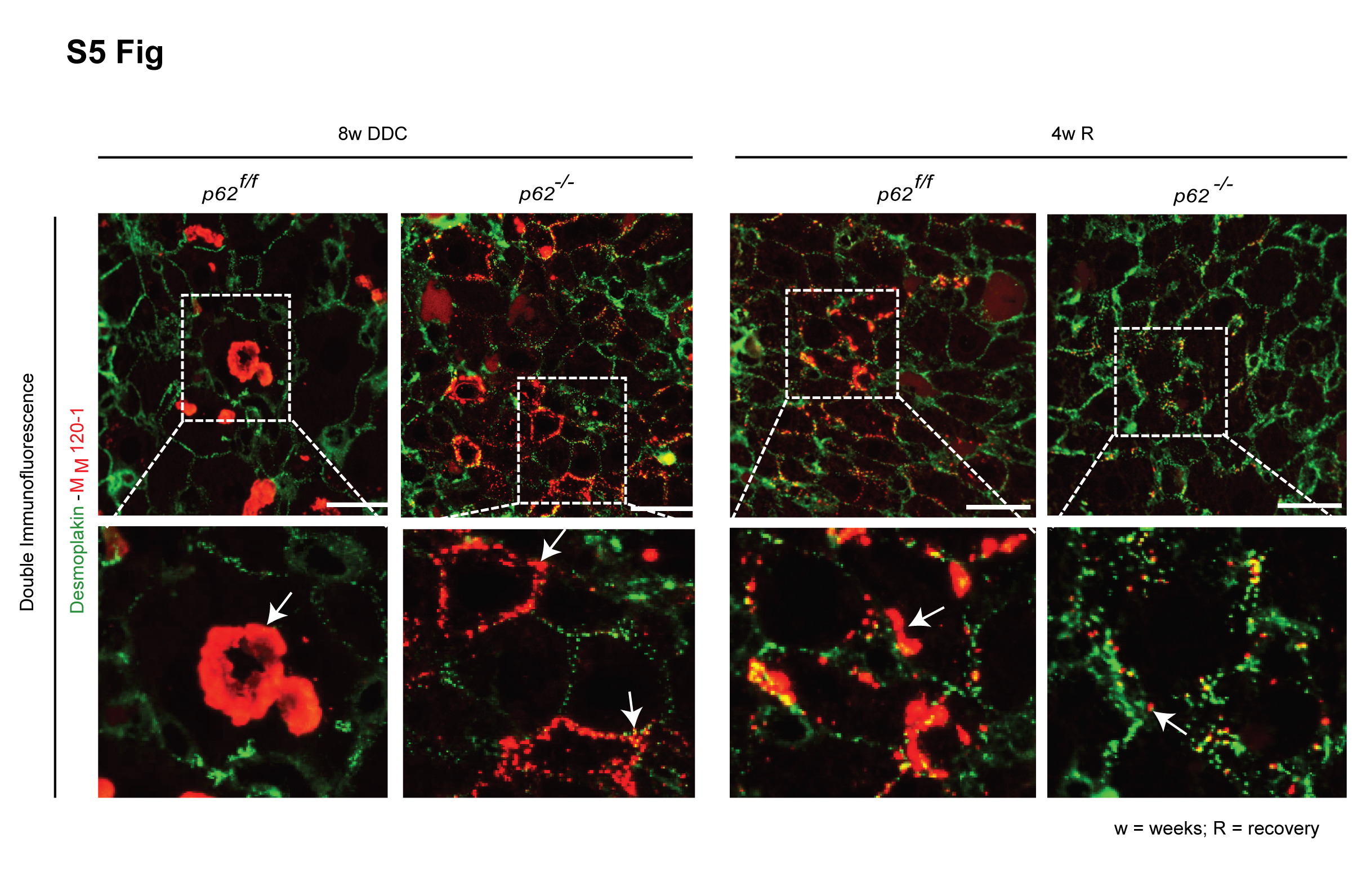

Supplement: S5 Fig — Double immunofluorescence staining with antibodies against MM120-1 (MDB marker; red) and desmoplakin (desmosomal marker; green) visualized the distribution of the antigens in 8 weeks DDC-intoxicated (8w DDC) and 4 weeks recovered (4w R) p62f/f and p62-/- mouse livers. MDBs are indicated by arrows. (Scale bar = 20 μm; inset showing higher magnification; scale bar = 10 μm). (TIF) [file pone.0161083.s005.tif]

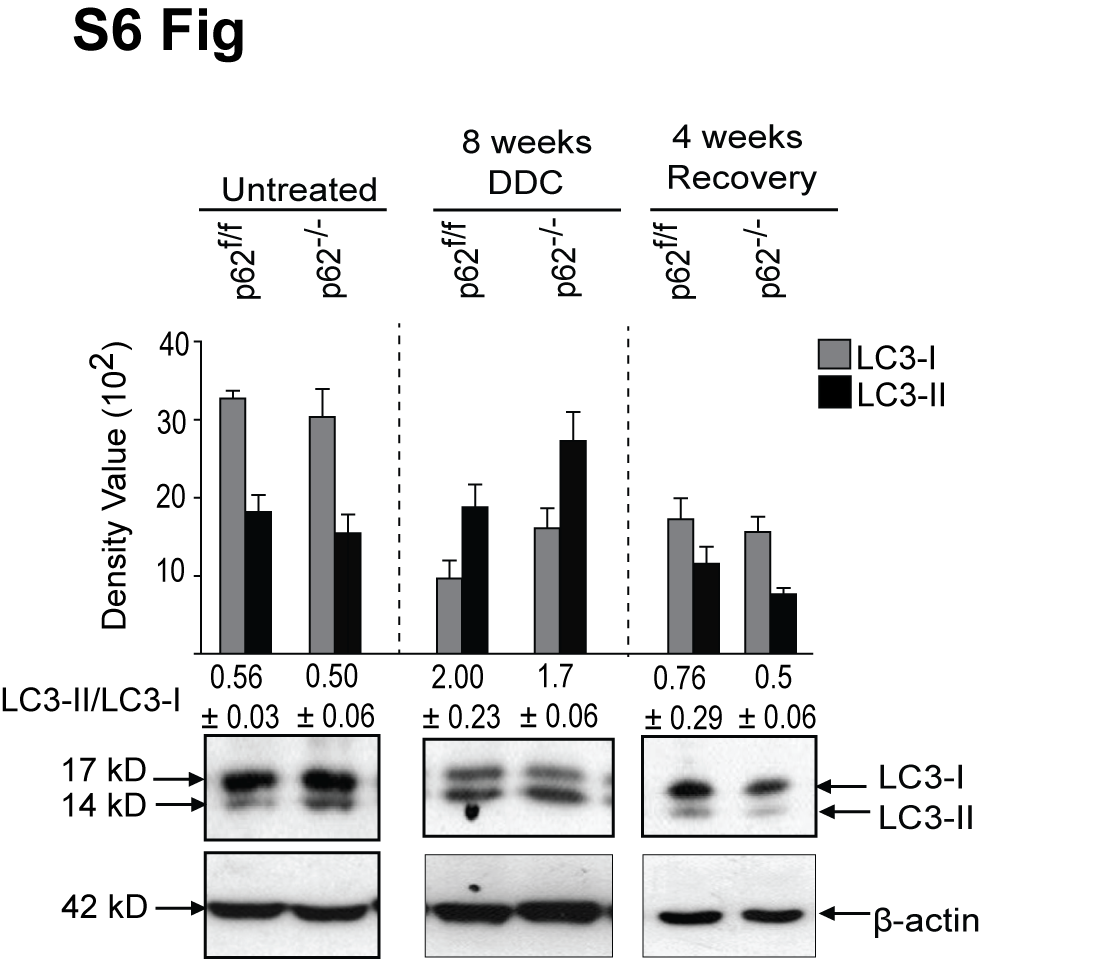

Supplement: S6 Fig — Whole tissue extracts from untreated mice, mice fed with DDC for 8 weeks (8w DDC) and from mice recovered on standard diet for four weeks (4w R) after DDC exposure were analyzed by western blotting with an antibody against LC3 as a marker of autophagy activation. Densitometric analysis depicted the intensity of both LC3-I and -II isoforms (dark/light bar) normalized to β-actin. DDC-intoxicated mice showed. higher LC3-II/I ratio but recovered mice acquired attenuation of autophagy (i.e. lower LC3-II/LC3-I ratio). The extent of autophagy did not differ between total p62-/- and p62f/f mice of the same treatment regimen. (TIF) [file pone.0161083.s006.tif]
