# Supplementary material for: p62/Sequestosome-1 Is Indispensable for Maturation and Stabilization of Mallory-Denk Bodies
Source: PLoS One. 2016 Aug 15;11(8):e0161083. doi: 10.1371/journal.pone.0161083 (PMC4985067; doi:10.1371/journal.pone.0161083)
Supplement: S1 Table — PCR A/B was performed to distinguish between p62 non-transgenic (p62NT), p62 floxed (p62f) and p62 ∆exon1-4 (p62- or p62hep-) while PCR C was performed to detect the presence of Cre-recombinase. (PDF) [file pone.0161083.s007.pdf]

Table S1. Genotyping PCR.

PCR	Forward Primer	Reverse Primer	*p62*^{NT}	*p62*^{f}	*p62*$^{-}$
A	CreA	CreB	154 bp	183 bp	
B	LoxPa	LoxPb			300 bp
C	CreF	CreR	380 bp	380 bp	380 bp

PCR A/B was performed to distinguish between *p62* non-transgenic (*p62*^{NT}), *p62* floxed (*p62*^{f}) and *p62*$^{\Delta exon1\text{-}4}$ (*p62*$^{-}$ or *p62hep*$^{-}$) while PCR C was performed to detect the presence of Cre-recombinase.
