# Supplementary material for: p62/Sequestosome-1 Is Indispensable for Maturation and Stabilization of Mallory-Denk Bodies
Source: PLoS One. 2016 Aug 15;11(8):e0161083. doi: 10.1371/journal.pone.0161083 (PMC4985067; doi:10.1371/journal.pone.0161083)
Supplement: S2 Table — (PDF) [file pone.0161083.s008.pdf]

Table S2. List of primers used for genotyping, qPCR and RT-PCR

Primer Name	Sequence
Genotyping Primers	
CreA	5' ATATCAGGGCCATAGGTGCTCA 3'
CreB	5' TGAGACAGGATCTTATACCACA 3'
LoxPa	5' TATCCTTACTAAACTGTATAGCCA 3'
CreF	5' ACCTGAAGATGTTCGCGATTATCT 3'
CreR	5' ACCGTCAGTACGTGAG- ATATCTT 3'
qPCR primers	
K8 Fwd	5' ATCAAGAAG GATGTGGACGA 3'
K8 Rev	5' CTGCAACTCACGGATCTC 3'
Nrf2 Fwd	5' CAGCATGATG GACTTGGAG 3'
Nrf2 Rev	5' AAATCCATGTCCTGCTGG 3'
Hmox-1 Fwd	5' ACATTGAGCTGTTTGAGGAG 3'
Hmox-1 Rev	5' GGTCTTTGTGTTCCTCTGTC 3'
Keap1 Fwd	5′ CATCCACCCTAAGGTCATGGA 3'
Keap1 Rev	5' GACAGGTTGAAGAACTCCTCC 3'
Nqo1 Fwd	5' GACCTTGCTTTCTATCACCA 3'
Nqo1 Rev	5' GATGCCACTCTGAATCGG 3'
Gst Fwd	5' GCTGACTTTGAGAAACGGA 3'
Gst Rev	5' CAAGGACATCATAGACGAGG 3'
β-actin Fwd	5' GTGACGAGGCCCAGAGCAAGAG 3'
β-actin Rev	5'AGGGGCCGGACTCATC GTACTC 3'
18S rRNA Fwd	5' GTAACCCGTTGAACCCCATT 3'
18S rRNA Rev	5' CCATCCAATCGGTAGTAGCG 3'
RT-PCR primers	
p62 Fwd	5' AAGTCAGCAAACCTGACG 3'
p62 Rev	5' CCATCTGTTCCTCTGGCT 3'
