# Supplementary material for: p62/Sequestosome-1 Is Indispensable for Maturation and Stabilization of Mallory-Denk Bodies
Source: PLoS One. 2016 Aug 15;11(8):e0161083. doi: 10.1371/journal.pone.0161083 (PMC4985067; doi:10.1371/journal.pone.0161083)
Supplement: S3 Table — (PDF) [file pone.0161083.s009.pdf]

Table S3. List of Antibodies used for immunofluorescence, immunohistochemistry and western blot

Antibody Name	Company
Primary antibodies	
p62	Progen; #GP62-C, Heidelberg, Germany
K8	Progen; #Ks 8.7, Heidelberg, Germany
K8	abcam; #ab53280, Cambridge, UK
K18	Progen; #Ks 18.04, Heidelberg, Germany
K18	abcam; #ab53118, Cambridge, UK
Ubiquitin	Cell Signalling; #3933S, Austria
Ubiquitin	Sigma Aldrich; #GW10073F, Vienna, Austria
NBR1	Novus; #NBP171703, Oxon, UK
M_M120-1	neat; (3)
LC3	Novus; #NB100-2200, Oxon, UK
Desmoplakin	abcam; #ab16434, Cambridge, UK
Secondary antibodies	
Alexa fluor goat anti rabbit IgG 488nm	Life technologies; #A11034, Vienna, Austria
Alexa fluor goat anti rabbit IgG 594nm	Life technologies; #A11012, Vienna, Austria
Alexa fluor goat anti mouse IgM 488nm	Life technologies; #A21042, Vienna, Austria
Alexa fluor goat anti mouse IgM 594nm	Life technologies; #A21044, Vienna, Austria
Alexa fluor goat anti chicken IgM 647nm	Life technologies; #A21449, Vienna, Austria
Goat anti-guineapig IgG HRP	DAKO; #P0141, Glostrup, Denmark
Goat anti-mouse IgG HRP	DAKO; #P0260, Glostrup, Denmark
